# The Role of NLRP3 Inflammasome in Alzheimer’s Disease and Potential Therapeutic Targets

**DOI:** 10.3389/fphar.2022.845185

**Published:** 2022-02-16

**Authors:** Tao Liang, Yang Zhang, Suyuan Wu, Qingjie Chen, Lin Wang

**Affiliations:** ^1^ Department of Clinical Laboratory, Union Hospital, Tongji Medical College, Huazhong University of Science and Technology, Wuhan, China; ^2^ Hubei Key Laboratory of Diabetes and Angiopathy, Medicine Research Institute, Xianning Medical College, Hubei University of Science and Technology, Xianning, China; ^3^ Research Center for Tissue Engineering and Regenerative Medicine, Union Hospital, Tongji Medical College, Huazhong University of Science and Technology, Wuhan, China

**Keywords:** Alzheimer’s disease, inflammation, neuroinflammation, NLRP3 inflammasome, mitochondrial dysfunction

## Abstract

Alzheimer’s disease (AD) is a common age-related neurodegenerative disease characterized by progressive cognitive dysfunction and behavioral impairment. The typical pathological characteristics of AD are extracellular senile plaques composed of amyloid *ß* (Aβ) protein, intracellular neurofibrillary tangles formed by the hyperphosphorylation of the microtubule-associated protein tau, and neuron loss. In the past hundred years, although human beings have invested a lot of manpower, material and financial resources, there is no widely recognized drug for the effective prevention and clinical cure of AD in the world so far. Therefore, evaluating and exploring new drug targets for AD treatment is an important topic. At present, researchers have not stopped exploring the pathogenesis of AD, and the views on the pathogenic factors of AD are constantly changing. Multiple evidence have confirmed that chronic neuroinflammation plays a crucial role in the pathogenesis of AD. In the field of neuroinflammation, the nucleotide-binding oligomerization domain-like receptor pyrin domain-containing 3 (NLRP3) inflammasome is a key molecular link in the AD neuroinflammatory pathway. Under the stimulation of Aβ oligomers and tau aggregates, it can lead to the assembly and activation of NLRP3 inflammasome in microglia and astrocytes in the brain, thereby causing caspase-1 activation and the secretion of IL-1β and IL-18, which ultimately triggers the pathophysiological changes and cognitive decline of AD. In this review, we summarize current literatures on the activation of NLRP3 inflammasome and activation-related regulation mechanisms, and discuss its possible roles in the pathogenesis of AD. Moreover, focusing on the NLRP3 inflammasome and combining with the upstream and downstream signaling pathway-related molecules of NLRP3 inflammasome as targets, we review the pharmacologically related targets and various methods to alleviate neuroinflammation by regulating the activation of NLRP3 inflammasome, which provides new ideas for the treatment of AD.

## 1 Introduction

Alzheimer’s disease (AD) is a common neurodegenerative disease that occurs in the elderly, and is also called senile dementia. The main clinical manifestations of AD patients are the progressive decline of self-care ability, cognitive impairment, and neuropsychiatric abnormalities, which seriously affects the quality of life of AD patients. The typical pathological features of AD are senile plaques related to extracellular amyloid *ß* (Aβ) deposition and neurofibrillary tangles formed by hyperphosphorylation of intracellular microtubule-associated tau (d'Errico and Meyer-Luehmann 2020). With the aging of the global society becoming more and more prominent, the increasing number of AD patients has become a major public health problem, which has brought a heavy burden to individuals, the society and families.

AD is first described by the German physician Alois Alzheimer in 1906 and has a history of more than one hundred years (Sanabria-Castro et al., 2017). The pathogenesis of AD is complex and diverse, which mainly involves genetic and environmental factors (Dunn et al., 2019), Aβ toxicity (Benilova et al., 2012), tau hyperphosphorylation (Wang et al., 2014), central nervous system (CNS) inflammation (Kinney et al., 2018), synaptic dysfunction (Li et al., 2018a), cholinergic deficiency (Frost et al., 2017), oxidative stress (Islam et al., 2019), mitochondrial dysfunction (Cardoso et al., 2004), autophagy and mitophagy abnormalities (Reddy and Oliver 2019), lipid metabolism disorder (Zhu et al., 2019), imbalance of calcium homeostasis (Popugaeva et al., 2015), endoplasmic reticulum (ER) stress (Huang et al., 2015), etc. Although the amyloid cascade hypothesis and the Tau protein theory are currently accepted by most investigators, the continuous and excessive neuroinflammatory response also plays a central role in the pathogenesis of AD. The nucleotide-binding oligomerization domain-like receptor pyrin domain-containing 3 (NLRP3) inflammasome is crucial in the neuroinflammatory pathway and has recently been highlighted as a potential target for AD treatment.

Inflammasome is a type of cytosolic multiprotein complex and plays a crucial role in innate immunity. The concept of inflammasome is first proposed by Tschopp and his colleagues in 2002. It is mainly composed of three parts: intracytoplasmic pattern recognition receptors (PRRs), the adaptor protein domain and the effector domain cysteine protease pro-caspase-1 (Martinon et al., 2002). In the CNS, the inflammasome mainly presents in the cytoplasm of immune cells, neuronal cells, microglia and astrocytes (Minkiewicz et al., 2013; von et al., 2018; Hanslik and Ulland 2020), and can recognize pathogen-associated molecular patterns (PAMPs) or host-derived danger-associated molecular patterns (DAMPs). Among the many reported inflammasomes, the NLRP3 inflammasome is currently the most studied one. Just like the structure of the above-mentioned inflammasomes, the NLRP3 inflammasome includes the sensor protein NLRP3, the adaptor protein apoptosis-associated speck-like protein containing a CARD (caspase activation and recruitment domain) (ASC), and the effector protein (pro-caspase-1, a cysteine protease) (Schroder and Tschopp 2010). These three proteins can interact closely to regulate the function of NLRP3 inflammasome. Once NLRP3 recognizes the foreign pathogen molecules or internal danger signals, it will be activated and undergos self-oligomerization. Then NLRP3 binds to the pyrin domain (PYD) domain of the adaptor protein ASC, and recruits the protease pro-caspase-1 to form the NLRP3 inflammasome, which cleaves pro-caspase-1 into activated caspase-1 through autocatalysis. The activated caspase-1, as an inflammasome effector protein, is able to cleave the inactive pro-inflammatory cytokines pro-IL-1β and pro-IL-18 into mature forms of IL-1β and IL-18, respectively. Ultimately, IL-1β and IL-18 are released outside of the cell to play a variety of non-specific inflammatory roles (Martinon et al., 2002; Kelley et al., 2019). In addition, the activated caspase-1 can also mediate a type of inflammatory-related programmed cell death, which is called pyroptosis. A large amount of inflammatory substances released after cell pyroptosis will induce a strong inflammatory response (Fink and Cookson 2006; Shi et al., 2015).

More and more experimental evidence show that the activation of NLRP3 inflammasome is closely related to neurodegenerative diseases (Duan et al., 2020; Feng et al., 2021). Under the stimulation of Aβ plaques and tau aggregates, microglia and astrocytes mediate chronic neuroinflammatory response, neuronal death and pyroptosis through intracellular NLRP3 inflammasome, thereby driving the occurrence and progression of AD (Han et al., 2020b; Van Zeller et al., 2021). More importantly, pharmacological inhibition of NLRP3 inflammasome exhibits neuroprotective effects. The use of inhibitory treatment against NLRP3 inflammasome can reduce Aβ deposition and alleviate the cognitive impairment of AD mice (Yan et al., 2020b). In this review, we mainly summarize the mechanisms of NLRP3 inflammasome activation, and analyze its possible roles in the progression of AD. In addition, we also introduce the upstream and downstream signaling pathways of the NLRP3 inflammasome, as well as the latest developments regarding its potential targets and therapeutic strategies for AD treatment.

## 2 The Activation and Regulation of NLRP3 Inflammasome

A certain number of exogenous or endogenous stimuli that induce the activation of NLRP3 inflammasome have been confirmed so far. The exogenous stimulating factors include lipopolysaccharide (LPS) (Ma et al., 2021), viral RNA (Allen et al., 2009), palmitate (Byeon et al., 2017), silica dioxide (Ko et al., 2020) and so on, while the damage-associated endogenous activators consist of ROS (Li et al., 2020a), cathepsin B (Bai et al., 2018), ATP (Amores-Iniesta et al., 2017), Aβ oligomers (Van Zeller et al., 2021), *α*-synuclein (*α*-syn) (Wang et al., 2020), etc. Although the process of NLRP3 inflammasome activation induced by the above factors has been extensively studied, the exact molecular mechanisms still need to be further explored. Current researches have shown that there are two main types of signaling pathways that are responsible for the activation of NLRP3 inflammasome. One is the canonical signaling pathway involving pro-caspase-1 recruitment and caspase-1 activation, and the other is the non-canonical signaling pathway, which is mainly related to the activation of mouse caspase-11 or human caspase-4 and caspase-5 induced by LPS.

### 2.1 Canonical NLRP3 Activation

As far as we know, the canonical NLRP3 inflammasome activation usually requires two steps: priming and activation (Yang et al., 2019). Generally speaking, in the resting state of cells, the basal levels of NLRP3 and IL-1β are considered to be insufficient to activate the inflammasome. Therefore, a priming step initiates the transcription of these targets. Priming signal (signal 1): NLRP3 is stimulated by danger signals (such as TLR4 agonists or endogenous molecules) to induce the expression of NF-κB, which up-regulates the transcription of NLRP3, IL-1β and IL-18 genes, resulting in the increased protein expression of NLRP3, pro-IL-1β and pro-IL-18 (Bauernfeind et al., 2009; Lamkanfi and Dixit 2014). Activation signal (signal 2): The second activation step is usually triggered by PAMPs or DAMPs (such as viral RNA, aluminum salt, ATP, Aβ, K^+^ efflux, etc.), which allows the NLRP3 inflammasome to complete the assembly step. Then, the cysteine protease pro-caspase-1 is recruited through the adaptor protein ASC to form a large filamentous protein complex called ASC speck. Clustered pro-caspase-1 autocatalyzes and autocleaves to generate activated caspase-1, which cleaves the pro-IL-1β and pro-IL-18 to generate the activated forms IL-1β and IL-18. At the same time, activated caspase-1 can initiate pyroptosis through the lysis of gasdermin D (GSDMD) (Swanson et al., 2019).

Recently, many studies have provided convincing evidence that the priming step of NLRP3 inflammasome activation is not limited to the increase of transcription level. There is another way to affect the activity of inflammasome through ubiquitination and post-translational modification of NLRP3. A recent study described that the recruitment of NEK7 to NLRP3 is controlled by the phosphorylation status of NLRP3 S803 located within the interaction surface, in which NLRP3 S803 is phosphorylated upon priming and later dephosphorylated upon activation. Phosphomimetic substitutions of NLRP3 S803 abolish NEK7 recruitment and inflammasome activity in macrophages *in vitro* and *in vivo* (Niu et al., 2021). Furthermore, Tang et al. found that E3 ubiquitin ligase TRIM65 can bind to the nucleotide-binding and oligomerization domain (NACHT) domain of NLRP3, promote lys48-and lys63-linked NLRP3 ubiquitination and inhibit NEK7-NLRP3 interaction, thereby restraining NLRP3 inflammasome assembly and caspase- 1 activation (Tang et al., 2021). In contrast, deubiquitination of NLRP3 leads to its activation. Studies have reported that the E3 ubiquitin ligase TRIM31 can directly bind to NLRP3 to promote K48-linked polyubiquitination and proteasomal degradation of NLRP3, thereby inhibiting the activation of NLRP3 (Song et al., 2016).

### 2.2 Non-Canonical NLRP3 Activation

In the non-canonical activation pathway, the NLRP3 inflammasome mainly relies on caspase-11 in mice (the homologues caspase-4 and caspase-5 in humans). LPS generated by Gram-negative bacteria enters the cytosol and can bind to caspase-11 in mice, thereby triggering its oligomerization and activation. The activated caspase-11 can induce pyroptosis and produce pro-inflammatory cytokines (Downs et al., 2020).

### 2.3 The Regulation of NLRP3 Activation

As summarized in previously published reviews, the main mechanisms involved in the activation of NLRP3 inflammasome include K^+^ efflux, cathepsin B released after lysosomal disruption, the change of extracellular Ca^2+^ homeostasis, and the production of reactive oxygen species (ROS) (Zhang et al., 2020), etc. We will not repeat any elaboration of the above activation mechanisms. However, in recent years, several direct or indirect ways have been reported to participate in the NLRP3 inflammasome activation. The latest studies have shown that mitochondrion is the central regulator of NLRP3 function. Mitochondrial reactive oxygen species (mtROS) production, mitochondrial DNA (mtDNA) release, mitochondrial-mediated apoptosis, mitochondrial calcium overload, and mitochondrial involvement in the localization of NLRP3 are all related to the regulation of NLRP3 activity (Zhou et al., 2011; Lawlor and Vince 2014; Rimessi et al., 2015). Therefore, we primarily discuss the roles of mitochondrial dysfunction, mitochondrial-associated endoplasmic reticulum membrane (MAM), autophagy and mitophagy in the activation and regulation of NLRP3 inflammasome in this review.

#### 2.3.1 Mitochondrial Dysfunction and NLRP3 Inflammasome Activation

Mitochondrion is one of the organelles with a double-layer membrane structure in cells, and it is the metabolic center and energy factory of cells. It provides the substrate and energy required for the biosynthesis of the cell, and plays a decisive role in the fate of cells. Mitochondria produce mtROS during aerobic metabolism via respiratory chain. Various intracellular and extracellular damage factors, including ROS, misfolded protein aggregation (such as Aβ, Tau, *α*-syn, etc.), toxic drugs, etc., can damage the normal function of mitochondria (Cha et al., 2012; Szabo et al., 2020). When the function of mitochondria is impaired, the level of mtROS increases significantly. mtROS accumulates in the cytoplasm and interacts with the components of the NLRP3 inflammasome, thereby participating in the activation of the inflammasome. In an earlier study, Nakahira et al. found that mtROS produced by impaired mitochondria is necessary for macrophages to activate NLRP3 inflammasome in response to LPS and ATP (Nakahira et al., 2011). Moreover, there is accumulating evidence to demonstrate that the use of chemical inhibitors to disrupt mitochondrial function can trigger the NLRP3 inflammasome activation. Mitochondrial dysfunction inducers such as rotenone (complex I inhibitor) can lead to increased levels of ROS, activation of NLRP3 inflammasome, and the expression of IL-1β in microglia (Sarkar et al., 2017). Furthermore, inhibitors or scavengers of mtROS can effectively restrain the activation of NLRP3 inflammasome. For example, the mtROS scavenger Mito-TEMPO inhibits the activation of NLRP3 inflammasome induced by injury factors and reduces the secretion of IL-1β (Ding et al., 2017). Consistent with these results, impaired clearance of damaged mitochondria will enhance the activation of NLRP3 inflammasome. However, there are also some inconsistent opinions about the relationship between mtROS and NLRP3 inflammasome activation. Some previous studies indicated that the activation of NLRP3 inflammasome may not depend on mtROS, but through other components of mitochondria (Iyer et al., 2013). Bauernfeind and his colleagues also showed that ROS inhibitors only blocked the priming step of NLRP3 inflammasome activation, while its direct activation step was not affected, which implied that the role of ROS was limited to the priming step of NLRP3 activation (Bauernfeind et al., 2011). Despite the existence of the above phenomenon, more and more evidence indicate that mtROS is located at upstream of NLRP3 inflammasome activation, and mtROS directly or indirectly participates in the process of NLRP3 inflammasome activation. Many drugs or chemical agents can alleviate the inflammatory effect of NLRP3 by reducing the level of mtROS.

Mitochondrial dysfunction causes increased mitochondrial breakage, which releases mtDNA, ATP, heat shock protein 60 (HSP60), mitochondrial transcription factor A (TFAM), cardiolipin, cytochrome c, etc. These substances can be considered as DAMPs to induce the activation of NLRP3 inflammasome (Dela and Kang 2018). Among them, mtDNA is the most extensively studied mitochondrial-derived activator. In an earlier study, Nakahira et al. showed that the release of mtDNA is crucial for the activation of NLRP3, which depends on the generation of ROS (Nakahira et al., 2011). Shimada et al. further used the 293 cells transfected with mtDNA to prove that mtDNA can directly bind to NLRP3 and mediate the activation of NLRP3 inflammasome. Conversely, macrophages lacking mtDNA severely reduce IL-1β production (Shimada et al., 2012). The increased levels of oxidized mtDNA (ox-mtDNA) in the cytoplasm can promote the binding with NLRP3 inflammasome, which leads to the co-localization of NLRP3 and ASC in the perinuclear space in endoplasmic reticulum-mitochondrial clusters (Zhong et al., 2018). Given that mtROS and ox-mtDNA are significantly related to the activation of NLRP3 inflammasome, a wide range of mitochondrial antioxidant drugs can attenuate the inflammasome activation. Epigallocatechin-3-gallate (EGCG) is a polyphenol with strong antioxidant properties. Luo et al. evaluated the protective effect of EGCG on acute pancreatitis (AP)-associated lung injury and found that EGCG could protect AP-associated lung injury by removing mtROS and its oxidation product ox-mtDNA. In addition, the antagonism of NLRP3 signaling by EGCG was affected in the presence of the mtROS stimulant rotenone or scavenger Mito-TEMPO (Luo et al., 2021). Idebenone is a highly acclaimed mitochondrial protective agent. In the oxygen glucose deprivation/reperfusion (OGD/R) injury model, Peng et al. found that mitochondrial dysfunction led to mtDNA translocation and mtROS production, as well as cytosolic accumulation of oxidized mtDNA, which promoted its binding to NLRP3. However, idebenone treatment effectively blocked this process, and alleviated NLRP3-mediated inflammatory damage after OGD/R (Peng et al., 2020). In short, increasing evidence show that mtDNA can be closely related to the expression of IL-1β through the NLRP3 inflammasome activation.

#### 2.3.2 The Regulation of NLRP3 Inflammasome Activation by Mitochondrial-Associated Endoplasmic Reticulum Membrane

The morphological structure of mitochondria and ER in eukaryotic cells is highly dynamic, which provides opportunities for coupling between mitochondria and ER. It has been reported that the mitochondrial outer membrane and the ER membrane can form an interaction coupling site membrane structure with a stable interval, which is known as MAM (Hayashi et al., 2009). In some places, MAM is also called mitochondria-ER contact sites (MERCs). MAM plays an important role in material transfer and signal transduction. At present, MAM has become a well-known important way for the regulation of cholesterol, lipids, calcium metabolism, oxidative stress, inflammation and other functions (Yu et al., 2021). The relationship between MAM and inflammation is discovered as early as 2011. In unstimulated cells, NLRP3 is mainly located on the ER membrane and in the cytoplasm. However, upon activation, NLRP3 and ASC will redistribute and translocate to the MAM in the perinuclear region, which makes it easier to sense mitochondrial damage signals such as mtROS, cardiolipin, mtDNA, etc (Zhou et al., 2011). MAM can be regarded as a platform for inflammasome assembly and activation. During the formation of inflammasome, acetylated *α*-tubulin can migrate mitochondria to the perinuclear region and promote the assembly of ASC on mitochondria with NLRP3 on the ER (Misawa et al., 2013). Recent studies have shown that MAM participates in the regulation of DAMPs-mediated effects, antiviral responses, bacterial pathogen-mediated infections, and other inflammatory processes through direct or indirect action (Missiroli et al., 2018). Martinvalet also has introduced the important role of mitochondria and the ER contact sites in the development of immune response (Martinvalet 2018). The NLRP3 on the ER and the ASC on the mitochondrial combine with each other through CARD to form the NLRP3 inflammasome, and those mitochondrial outer membrane proteins involved in ER-mitochondrial binding, such as mitogen, can regulate the structural stability of MAM, thereby controlling the activation of NLRP3 inflammasome. Mitochondrial antiviral signal protein (MAVS) is an adaptor molecule located on the outer mitochondrial membrane, which participates in the secretion of type I interferon. As an important component of MAM, it plays a pivotal role in regulating the host’s natural immunity (Horner et al., 2015). Studies found that MAVS can recruit NLRP3 to mitochondria in response to viral infections. MAVS is linked to the N-terminal amino acid sequence of NLRP3, which is the basis of interaction between MAVS and NLRP3 (Subramanian et al., 2013). In addition, a study carried out by Guan et al. proved that MAVS is capable of stabilizing ASC and inducing the formation of cytosolic speck via recruiting the E3 ligase TRAF3 to ASC. Ubiquitination of ASC at Lys174 by TRAF3 is essential for speck formation and inflammasome activation. The deficiency of MAVS or TRAF3 will impair ASC ubiquitination and the formation of cytoplasmic speck, thereby reducing the NLRP3 inflammasome activation (Guan et al., 2015). Mitofusin 2 (MFN2) is a mitochondrial outer membrane GTPase, which plays an important role in the mitochondrial fusion process. Furthermore, MFN2 is also present on the ER membrane. MFN2 is enriched in MAM and enhances the structural stability of MAM. MFN2 on the ER bridges ER and mitochondria by engaging in homotypic and heterotypic complexes with mitofusin 1 or 2 on the surface of mitochondria (de Brito and Scorrano 2008). The stable MAM structure may provide a basis for the assembly of NLRP3 inflammasome. An earlier study showed that after infection with influenza virus or encephalomyocarditis virus (EMCV), MFN2 could interact with NLRP3 to promote the recruitment of NLRP3 to mitochondria, and subsequently induce IL-1β secretion. However, the secretion of IL-1β was significantly restored in MFN2 gene knockout cells (Ichinohe et al., 2013). Another study described that infection with *mycobacterium tuberculosis* up-regulated the expression of MFN2 and promoted the assembly and activation of the NLRP3 inflammasome (Xu et al., 2020). These researches imply that MFN2 may contribute to the stability of MAM structure, and promote the activation of NLRP3 inflammasome. However, the specific mechanism still needs further studies.

It is generally acknowledged that Ca^2+^ play an important role in NLRP3 inflammasome activation (Horng 2014). ER is the main Ca^2+^ reservoir in cells. The continuous transfer of Ca^2+^ from ER into the mitochondria will result in mitochondrial Ca^2+^ overload and dysfunction, which promotes the release of cardiolipin and mtDNA (Murakami et al., 2012). MAM is the main site that mediates the transportation of Ca^2+^ from ER to mitochondria, which is related to the distribution of Ca^2+^ transport channel proteins in the MAM region. The IP3R-GRP75-VDAC-MCU complex is a classic pathway that mediates the transport of ER Ca^2+^ to the mitochondria through the MAM region (Szabadkai et al., 2006). These proteins are also the constituent molecules of MAM. Inhibitors or gene knockouts against these molecules may attenuate NLRP3 inflammasome activation. We believe that the changes of MAM function will affect the activation of NLRP3 inflammasome. Therefore, drugs or compounds that cause changes in MAM function can regulate the NLRP3 inflammasome activation.

#### 2.3.3 The Negative Regulation of NLRP3 Inflammasome via Autophagy and Mitophagy

Autophagy is a process of non-specific degradation of the cell’s own components such as organelles and abnormal accumulation proteins through the lysosomal system. Hence, it is essential for maintaining cell homeostasis and survival (Mameli et al., 2021). Autophagy has been confirmed to be closely related to the NLRP3 inflammasome activation, as the response of eukaryotic cells to external stimuli. In an earlier study, Saitoh Tatsuya et al. reported that the important autophagy gene Atg16L1 regulated endotoxin-induced inflammasome activation. In LPS-stimulated macrophages, the deficiency of Atg16L1 could lead to activation of NLRP3 inflammasome and production of IL-1β (Saitoh et al., 2008). Furthermore, Atg5 is also an important autophagy-related gene. Atg5 acetylation can inhibit the maturation of autophagosomes and induce the activation of NLRP3 inflammasome. On the contrary, sirtuin 3 (SIRT3) can form a complex with Atg5 to block the acetylation of Atg5, which leads to impaired autophagy and accelerates the activation of NLRP3 inflammasome (Liu et al., 2018). As far as we know, there is mounting evidence show that autophagy is an important regulator of inflammasome, which negatively regulate the NLRP3 inflammasome activation. Autophagy can eliminate the endogenous activator DAMPs. In AD, autophagy alleviates the activation of NLRP3 inflammasome induced by Aβ oligomers via removing abnormally deposited and misfolded proteins (Wen et al., 2019). Mi-Hyang Cho et al. revealed that, in the microglia model, Aβ interacts with MAP1LC3B-II through OPTN/optineurin and is degraded by the autophagy process mediated by the PRKAA1 pathway (Cho et al., 2014). Deficiency or inhibition of autophagy can exacerbate the pathology of NLRP3 inflammasome-mediated neurodegenerative diseases (Qin et al., 2021). In contrast, autophagy inducers, such as rapamycin, AICAR, and metformin, can activate autophagy in microglia, which promotes the phagocytosis and degradation of misfolded protein aggregates in cells, thereby effectively inhibiting the excessive activation of NLRP3 inflammasome (Qiu et al., 2020).

Mitophagy is a process that selectively removes damaged mitochondria. Once mitochondrial dysfunction occurs, mitophagy can promote the renewal of mitochondria, thereby maintaining mitochondrial quality control. As mentioned above, there is growing evidence that damaged mitochondria activate the NLRP3 inflammasome through a variety of ways. Therefore, mitophagy can be considered as an important way to regulate the activation of NLRP3 inflammasome. Currently, multiple literatures demonstrate that mitophagy also negatively regulates the NLRP3 inflammasome activation. Mitophagy eliminates damaged mitochondria, avoids the release of endogenous molecules such as ATP, mtROS and mtDNA, thus reduces the activation of NLRP3 inflammasome (Mishra et al., 2021). Mitophagy inhibitors or gene knockouts can lead to mitophagy disorder, cause the accumulation of mtROS and mtDNA in cells, and activate the NLRP3 inflammasome. Researching the role of Parkin, a central player in mitophagy, in host antiviral responses, Li et al. found that Parkin deficiency augments innate antiviral inflammation and promotes viral clearance by enhancing mtROS-mediated NLRP3 inflammasome activation (Li et al., 2019). On the contrary, mitophagy inducers can enhance the ability to clear dysfunctional mitochondria, thereby inhibiting NLRP3 inflammasome activation (Peng et al., 2021). Gao et al. reported in the nonalcoholic fatty liver disease (NAFLD) model that the expression levels of mitophagy markers PINK1 and Parkin was significantly diminished by deoxycholic acid (DCA) and the ability of mitophagy was impaired. However, after treatment with a specific mitophagy agonist carbonyl cyanide 3-chlorophenylhydrazone (CCCP), the ability of mitophagy was restored and the DCA-induced inflammasome response was prevented (Gao et al., 2021b). In conclusion, numerous current studies have shown that autophagy and mitophagy may be a self-limiting way to protect cells from excessive inflammation.

## 3 The Role of NLRP3 Inflammasome in Alzheimer’s Disease

Neuroinflammation is a double-edged sword. It is regarded as a defensive mechanism during the acute infection period and plays an anti-infection role. However, after its transfer to the chronic inflammation phase, excessive release of cytotoxic factors will cause inflammation activation. Increasing evidence from AD patients, *in vitro* cell models and *in vivo* animal models indicate that NLRP3 inflammasome plays an important role in AD. Saresella et al. showed that the expression level of NLRP3 inflammasome-related molecules was higher in severe AD patients than moderate ones via gene expression analysis of peripheral blood mononuclear cells (PBMCs) in AD patients. *In vitro* stimulation of PBMCs with LPS or Aβ42 could activate NLRP3 inflammasome. They believe that peripheral monocytes are likely to migrate across the blood-brain barrier (BBB) into the CNS and participate in the neuroinflammatory response of AD (Saresella et al., 2016). Mahmoudiasl et al. further detected increased expression levels of NLRP3, caspase-1, and inflammasome activation products IL-1β and IL-18 in the cerebral temporal cortex of AD patients (Ahmed et al., 2017). Aβ fibrils have unique structural characteristics and can be regarded as a kind of DAMPs, which are recognized by Toll-like receptors (TLRs) or nucleotide-binding oligomerization domain-like receptors (NLRs) and transmit pro-inflammatory signals. Early studies reported that the senile plaques are surrounded by activated microglia and astrocytes, and the glial cells around the Aβ plaques express higher levels of IL-1β (Apelt and Schliebs 2001). Subsequently, Halle et al. first described the role of NLRP3 inflammasome in the AD model. They found that Aβ activates the NLRP3 inflammasome in microglia, causing the maturation and secretion of IL-1β and IL-18. The increased amount of Aβ phagocytosed by microglia can cause lysosomal damage in the cytosol and the release of cathepsin B, and the latter can act as an endogenous danger signal to activate the NLRP3 inflammasome (Halle et al., 2008). Recent studies have shown that NLRP3 inflammasome is not only activated by fibrous Aβ aggregates, but also by lower molecular weight Aβ oligomers and fibrils. This suggests that the innate immune response of CNS triggered by Aβ activation may be before the onset of Aβ deposition (Luciunaite et al., 2020). The researchers further have explore the mechanisms by which Aβ activates the NLRP3 inflammasome and have found that this may involve two signals: the priming signal and the activation signa. When studying the inflammatory response of primary microglia to Aβ (1–42) protofibrils, Terrill-Usery et al. found that Aβ (1–42) protofibrils significantly upregulates the expression of IL-1β, TNFα mRNA and pro-IL-1β protein through the TLR/MyD88 pathway (Terrill-Usery et al., 2014). Similarly, the results of Liu et al. showed that Aβ(1–42) activates and up-regulates the expression of NLRP3 inflammasome-related molecules in BV-2 microglia via the TLR4/NLRP3 pathway and increases the secretion of IL-1β (Liu et al., 2020). These results indicate that Aβ fibrils can provide the priming signal for NLRP3 inflammasome activation. Another study revealed that Aβ induces the formation of NLRP3 inflammasome in a cathepsin-dependent manner. Under resting conditions, NLRP10 can bind to ASC and inhibit the assembly of NLRP3 inflammasome. However, after glial cells are treated by Aβ, cathepsin can be activated to promote the degradation of NLRP10, which makes it easier for NLRP3 and ASC to combine with each other to form inflammasomes (Murphy et al., 2014). This indicates that Aβ fibrils can also provide activation signals for NLRP3 inflammasome in an indirect way. In short, the above evidence mainly reflect that Aβ activates the NLRP3 inflammasome, and then participates in the pathogenesis of AD through IL-1β, IL-18 and other inflammatory cytokines. Moreover, it has been proposed that Aβ1-42 can also mediate GSDMD lysis through NLRP3-caspase-1 signal, and induce neuronal cell pyroptosis (Han et al., 2020).

In recent years, a large amount of data from cell experiments and animal models have confirmed that the activation of NLRP3 inflammasome can also affect the deposition and spread of Aβ. Heneka et al. found that, compared with APP/PS1 mice, NLRP3 and caspase-1 knockout AD model mice have a significantly enhanced ability of microglia to phagocytose Aβ and differentiate microglia into anti-inflammatory M2 type, which facilitates Aβ clearance (Heneka et al., 2013). In addition, the ability of microglia to clear Aβ can also be enhanced by inhibitors of NLRP3 or caspase-1, thereby reducing the accumulation of Aβ in the brains of APP/PS1 mice (Dempsey et al., 2017). These results confirm that the activation of NLRP3/caspase-1 inflammasome reduces the phagocytosis of Aβ by glial cells, which makes it easier for Aβ to accumulate in the cells. After comprehensive analysis of the related research results of Aβ and NLRP3 inflammasome, we speculate that when Aβ oligomers or fibrils activate NLRP3 inflammasome, it regulates the production of neurotoxic inflammatory cytokines such as IL-1β and IL-18. At the same time, it can induce pyroptosis of neurons by activating caspase-1 to mediate the lysis of GSDMD. On the other hand, the activation of NLRP3 inflammasome can conversely lead to increased Aβ deposition and diffusion in glial cells, thereby inducing Aβ into the positive feedback loop, and ultimately contributing to the development of AD.

**FIGURE 1:**
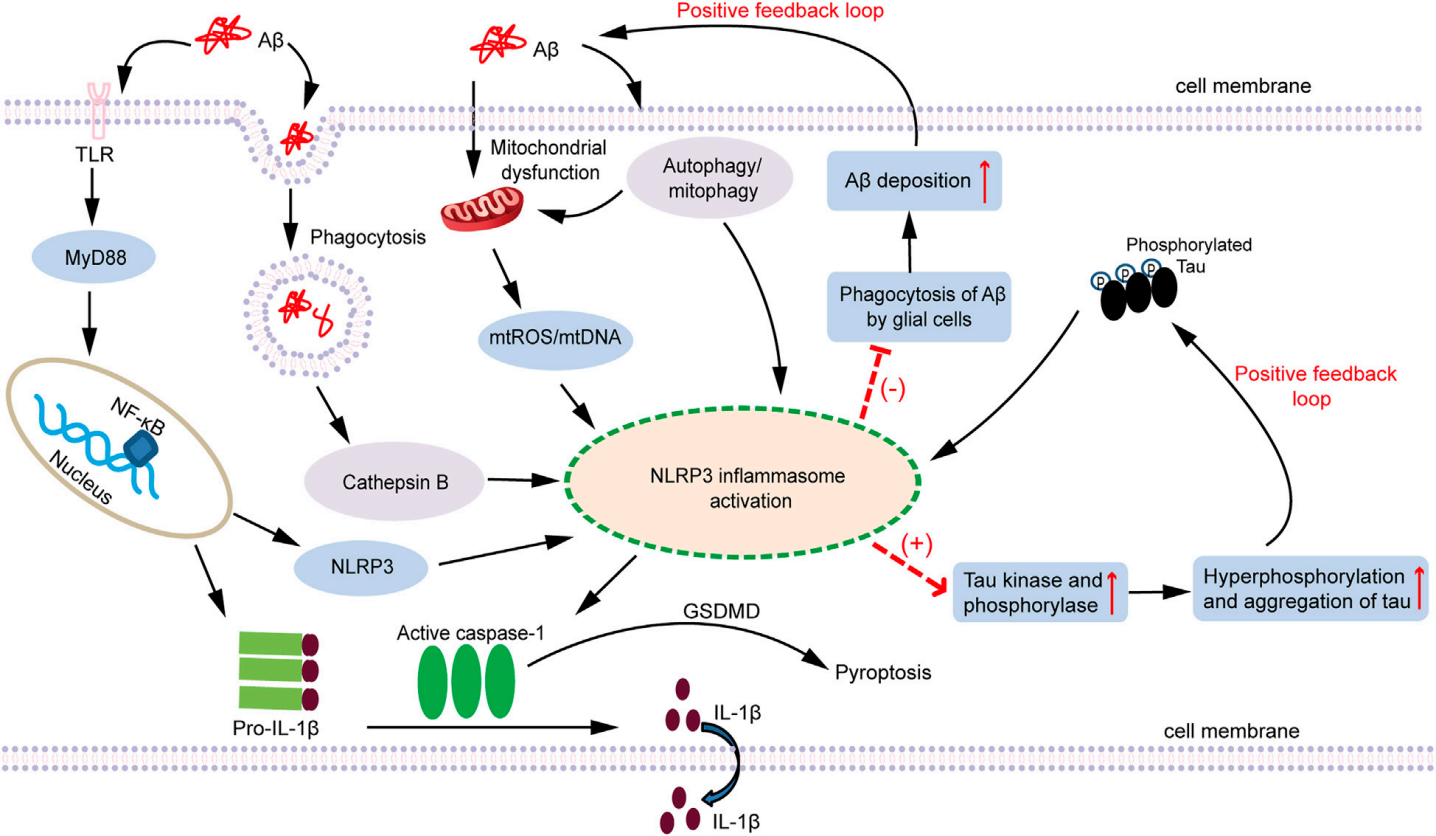
A schematic diagram of the association between NLRP3 inflammasome activation and AD pathogenesis. Both Aβ oligomers and Tau aggregates are involved in the inflammatory response of AD. Fibrillar Aβ species are regarded as PAMPs that triggers NF-κB activation through pattern recognition receptors (such as TLRs) to elevate inflammasome components NLRP3 and pro-IL-1β. NLRP3, ASC, and pro-caspase-1 assemble together to form the NLRP3 inflammasome, which subsequently activates caspase-1, cleaves pro-IL-1β to produce the active form of IL-1β and secretes it extracellularly. In addition, phagocytosis of soluble Aβ also triggers lysosome leakage and consequently results in the emission of cathepsin B, which leads to NLRP3 inflammasome activation. Furthermore, Aβ oligomers can act as damaging stimulis to induce mitochondrial dysfunction, causing the production and accumulation of ROS, release of mtDNA, or cardiolipin externalization, which activates the NLRP3 inflammasome. Autophagy can not only clear Aβ, but also clear NLRP3, ASC and pro-caspase-1 inflammasome-related protein molecules. Mitophagy can selectively remove impaired mitochondria and relieve the release of damaging molecules within mitochondria. Therefore, autophagy and mitophagy can negatively regulate the activation of NLRP3. Aβ also indirectly regulate the activation of the NLRP3 inflammasome through the autophagy or mitophagy pathway. Moreover, the activation of NLRP3 inflammasome inhibits the phagocytosis of Aβ by glial cells, which contributes to the deposition of Aβ and facilitates the formation of Aβ plaques. In conclusion, Aβ can activate the NLRP3 inflammasome through different pathways. However, once the NLRP3 inflammasome is activated, it in turn increases the deposition of Aβ and the formation of Aβ plaque, which forms a positive feedback loop that amplifies Aβ pathogenic effect. Similar to the role of Aβ, Tau is regarded as an endogenous dangerous molecule that can activate the NLRP3 inflammasome. After the NLRP3 inflammasome is activated, it increases the activity of Tau kinase and phosphorylase, and facilitates the phosphorylation and aggregation of Tau, thereby also forming a positive feedback loop. Persistent activation of the NLRP3 inflammasome triggered by Aβ and Tau contributes to the development of chronic neuroinflammation, which ultimately leads to the neuronal loss and cognitive impairment. AD: Alzheimer’s disease; Aβ: amyoid β; PAMPs: pathogen-associated molecular patterns; TLRs: Toll-like receptors; NF-κB: nuclear factor kappa B; NLRP3: nucleotide-binding oligomerization domain-like receptor pyrin domain-containing 3; ASC: apoptosis-associated speck-like protein containing a CARD; ROS: reactive oxygen species; mtROS: mitochondrial ROS; mtDNA: mitochondrial DNA; MyD88: myeloid differentiation factor 88; GSDMD: gasdermin D.

Although there are many researches to reveal the role of Aβ aggregates in the activation of NLRP3 inflammasome, there are limited studies on the relationship between Tau and NLRP3 inflammasome. In an earlier study, Kitazawa et al. used IL-1R blocking antibodies to inhibit IL-1β signaling in the 3xTg AD mouse model and found that they could significantly reduce the activity of tau kinase, such as cdk5/p25, GSK -3β, p38-MAPK, thereby reducing the level of tau phosphorylation (Kitazawa et al., 2011). This implies that the inflammatory effect after activation of NLPR3 inflammasome may have an impact on the pathogenic effect of Tau. In 2019, a major study revealed the influence of NLPR3 inflammasome on the pathology of tau. Ising et al. found that the loss of NLRP3 function could reduce the hyperphosphorylation and aggregation of tau by regulating tau kinase and phosphorylase. In addition, intracerebral injection of homogenate containing Aβ fibrils induced pathological changes of tau protein, which depends on the activation of NLRP3. Their study confirms that the activation of NLPR3 inflammasome in microglia plays an important role in the pathological changes of tau. Meanwhile, it also supports the Aβ cascade hypothesis in the pathogenesis of AD and the role of neurofibrillary tangles in the downstream development of Aβ-induced activation of microglia (Ising et al., 2019). In the same year, another highlighted study investigated whether Tau aggregates could activate NLRP3 inflammasome just like Aβ fibrils. They demonstrated that Tau activates NLRP3 inflammasome after being taken up by microglias, and its activation mechanism is similar to that of Aβ. Moreover, Tau-induced pathology is alleviated in Tau transgenic mice with ASC gene deletion or NLRP3 targeting inhibitors (Stancu et al., 2019). Surprisingly, tau protein may also provide the priming signal for the activation of NLRP3 inflammasome. Panda et al. used tau-derived PHF6 peptide (VQIVYK) to stimulate microglia and found that VQIVYK in the form of fibrous aggregates upregulated the expression of NLRP3 at mRNA and protein levels in a dose- and time-dependent manner, ultimately leading to increased expression of IL-1β and IL-18 (Panda et al., 2021). Experimental results from *in vivo* also show that hyperphosphorylation of tau in the mouse brain significantly increases the activation of NLRP3 inflammasome and the up-regulation of IL-1β levels (Zhao et al., 2021). In summary, we speculate that the role of tau in NLRP3 inflammasome is similar to Aβ. On the one hand, tau aggregates activate the NLPR3 inflammasome to regulate the expression and secretion of IL-1β and IL-18 and participate in the pathological damage of tau. On the other hand, the activation of NLRP3 inflammasome can also increase the hyperphosphorylation and aggregation of tau through tau kinase and phosphorylase, thereby inducing tau to go through the positive feedback loop, and ultimately playing an important role in the pathogenesis of AD (Figure 1).

## 4 NLRP3 Inflammasome Inhibitors as a Potential Target for the Treatment of Alzheimer’s Disease

**FIGURE 2:**
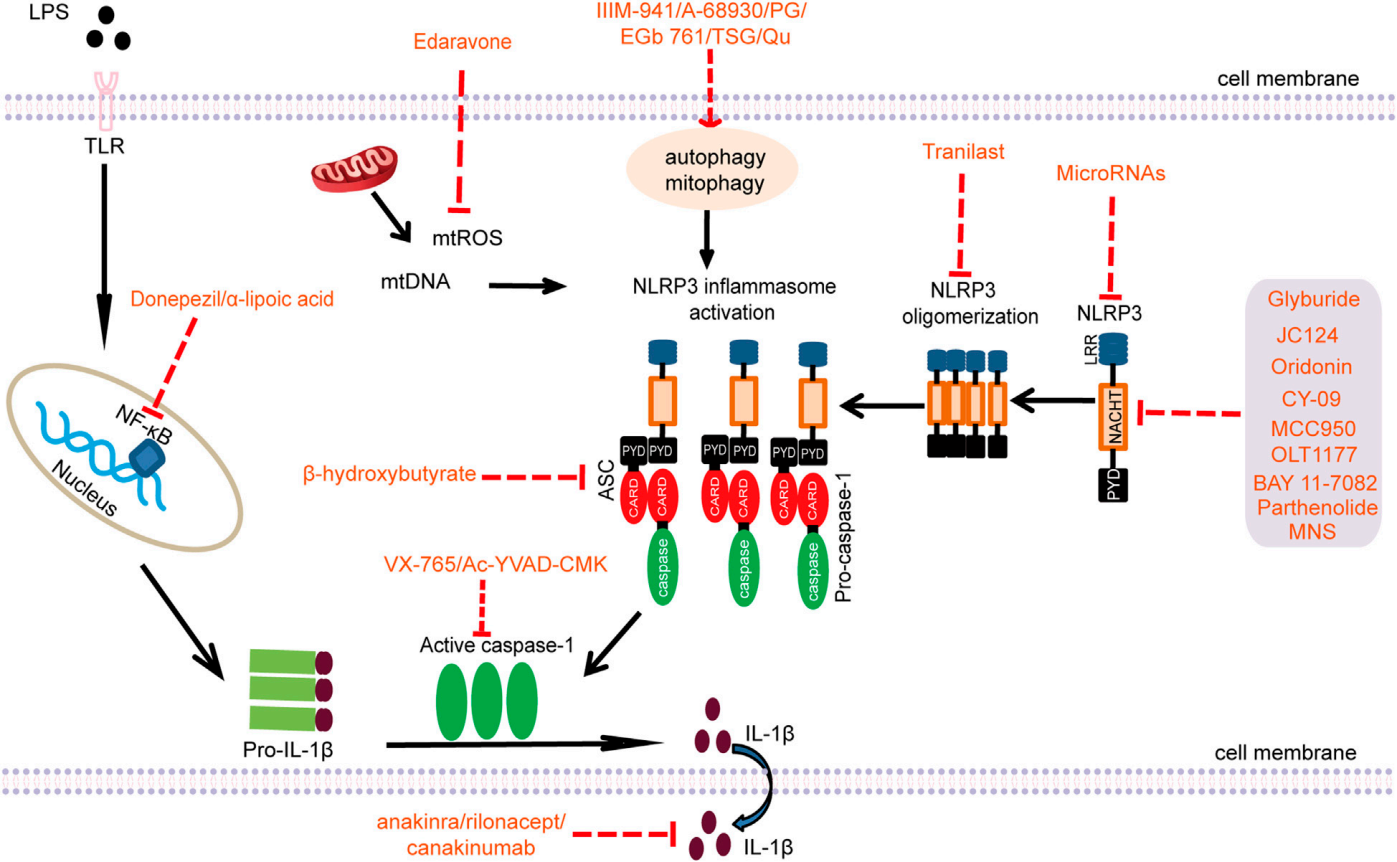
Pharmacological targets of the NLRP3 inflammasome (LPS: lipopolysaccharide; TLR: Toll-like receptor; NF-κB: nuclear factor kappa B; mtROS: mitochondrial ROS; mtDNA: mitochondrial DNA; NLRP3: nucleotide-binding oligomerization domain-like receptor pyrin domain-containing 3; ASC: apoptosis-associated speck-like protein containing a CARD; PG: progesterone; TSG: 2,3,5,4′-Tetrahydroxystilbene-2-O-β-D-glucoside; Qu: Quercetin; MNS: 3,4-methylenedioxy-β-nitrostyrene; CARD: caspase recruitment domain; NACHT: nucleotide-binding and oligomerization domain; PYD: pyrin domain; LRR: leucine-rich repeat).

With the comprehensive understanding of the molecular mechanism of NLRP3 inflammasome activation, since 2013, many published articles have paid more attention to the therapeutic value of targeted intervention of NLRP3 inflammasome in diseases. In view of the important role of NLRP3 inflammasome in the pathogenesis of AD, exploring its drug targets in the treatment of AD has also become a hot topic in its field. According to the characteristics of the formation and activation of NLRP3 inflammasome, some compounds that inhibit the activity of NLRP3 or interfere with its interaction with ASC are used to block the activation of NLRP3 inflammasome, which provides new ideas for the treatment of AD. Furthermore, considering the secretion of inflammatory factors downstream of the NLRP3 inflammasome and pyroptosis, the targeted intervention of caspase-1 activation and inhibition of downstream inflammatory factors of NLRP3 may also be a way to alleviate chronic inflammation in AD. The NLRP3 inflammasome and involvement of several upstream or downstream signaling pathways provide promising pharmacological targets for AD (Figure 2). At present, in the strategy of AD treatment, some compounds that directly inhibit the activity of NLRP3 ATPase include CY-09, MNS and OLT1177. There are some drugs interfering with ASC oligomerization, which is represented by *ß*-hydroxybutyric acid (BHB). The inhibitors of caspase-1 activation are VX-740 and VX-765. Biological agents targeting IL-1β mainly include IL-1β antibody canakinumab and recombinant IL-1β receptor antagonist anakinra. In the following section, we will review and summarize in detail the role and therapeutic value of the above interventions in AD (Table 1).

**TABLE 1:** The compounds or extractions targeting NLRP3 inflammasome pathways in AD.

Compounds or extractions	Mechanism	Cell or animal model	References
IL-1 inhibitors			
anakinra	IL-1 receptor antagonist	3xTg-AD transgenic mice	Kitazawa et al. (2011)
	IL-1 receptor antagonist	AD amyloidosis rat model	Qi et al. (2018), Batista et al. (2021)
rilonacept	IL-1inducible receptor	—	Giancane et al. (2016)
canakinumab	Antibody targeting IL-1β	—	Giancane et al. (2016)
NLRP3 inhibitors			
Glyburide	ATP sensible K^+^ channels, downstream of the P2X7 receptor	—	Lamkanfi et al. (2009)
JC124	Inhibits the NLRP3 inflammasome and the activation of caspase-1	APP/PS1 or CRND8 APP transgenic mice	Fulp et al. (2018), Yin et al. (2018), Kuwar et al. (2021)
Oridonin	Covalent bond with NLRP3 in NACHT domain to block the interaction between NLRP3 and NEK7	Aβ_1-42_ induced AD mice	Wang et al. (2014), Wang et al. (2016), He et al. (2018)
CY-09	Binds to the ATP binding motif of the NLRP3 NACHT domain to inhibit NLRP3 ATPase activity	—	Jiang et al. (2017)
MCC950	Walker B motif interaction and inhibition of ATP hydrolysis, selective inhibitor of NLRP3	APP/PS1 AD, Long evans rats, SAMP8 mouse	Qi et al. (2018), Coll et al. (2019), Fekete et al. (2019), Li et al., 2020b)
	Selective inhibitor of NLRP3	Microglia induced by Aβ aggregates	Luciunaite et al. (2020)
OLT1177	Binds to NLRP3 to inhibit its ATPase activity	APP/PS1 mice	Marchetti et al. (2018), Lonnemann et al. (2020)
Tranilast	Directly binds to the NACHT of NLRP3 and blocks NLRP3 oligomerization	—	Huang et al. (2018)
BAY 11–7082	Inhibits NLRP3 ATPase activity	APP 23 mice, BV2 cells	Ruan et al. (2019)
Parthenolide	Inhibits NLRP3 ATPase activity and caspase-1	Primary glial cells	Ou et al. (2020)
MNS	Inhibits the activity of NLRP3 ATPase through binding to the LRR and NACHT domains	—	He et al. (2014)
ASC inhibitors			
BHB	Prevents K^+^ efflux and reduces ASC oligomerization and speck formation	5xFAD mouse	Youm et al. (2015), Shippy et al. (2020)
	Improves the cognitive function	AD patients	Ota et al. (2019)
Caspase-1 inhibitors			
VX-765	Inhibits caspase-1	AD J20 mouse	Flores et al. (2018), Flores et al. (2020)
Ac-YVAD-CMK	Inhibits caspase-1	APP/PS1 AD mice	Gu et al. (2021)
Plant-derived compounds			
Resveratrol	Inhibits TXNIP/TRX/NLRP3 signaling pathway	BV-2 cells	Feng and Zhang, (2019)
	Inhibits NF-κB/IL-1β/NLRP3 signaling pathway	AD mouse model induced by Aβ_1-42_	Qi et al. (2019)
Pterostilbene	Inhibits the NLRP3/caspase-1 pathway	Microglia induced by Aβ_1-42_	Li et al. (2018)
SFN	Inhibits the NLRP3 inflammasome	N9 microglial cells	Tufekci et al. (2021)
GB	Inhibits NLRP3 activation and promotes microglia M2 polarization	BV2 microglial cells induced by Aβ_1-42_	Zhang et al. (2021b)
ABPPκ	Inhibits the expression of NLRP3, cleaved caspase-1, and ASC	BV2 microglia, Aβ oligomers-injected mice	Ge et al. (2021)
Chinese herbal medicines			
PK	Inhibits the NLRP3 inflammasome	5xFAD mouse	Kim et al. (2020)
DHM	Inhibits the NLRP3 inflammasome	APP/PS1 mice	Feng et al. (2018)
NSAIDs			
IND	Reduces the expression of IL-1β and caspase-1	AD rats induced by streptozotocin	Karkhah et al. (2021)
MicroRNAs	Directly or indirectly inhibits the expression of NLRP3	Glial cells, AD mice, and AD patients	Han et al. (2020), Feng et al. (2021), Wan et al. (2021)
Autophagy activators			
A-68930	Enhances the degradation of NLRP3 inflammasome by activating the AMPK/autophagy signaling pathway	BV2 cells, AD mice induced by Aβ_1-42_	Cheng et al. (2020)
PG	Inhibits the activation of NLRP3-caspase-1 via enhancing the autophagy	Astrocytes	Hong et al. (2019)
EGb 761	Down-regulates the level of NLRP3 protein, reduces the activation of IL-1β and caspase-1 via autophagy	TgCRND8 AD model	Liu et al. (2015)
Mitophagy activators			
TSG	Prevents NLRP3 inflammation through mitophagy	APP/PS1 mice, BV2/N2a/SH-SY5Y cells	Gao et al. (2020)
Qu	Inhibits NLRP3 inflammation through mitophagy	Primary microglia, BV2 cells	Han et al. (2021)
ROS and NF-κB inhibitors			
*α*-lipoic acid	Inhibits NLRP3 via the NF-κB signaling pathway	BV-2 microglial cells	Kim et al. (2019)
Edaravone	Reduces the production of mtROS, and inhibits the activation of NLRP3	Aβ-treated microglia	Wang et al. (2017)
Donepezil	Down-regulates NLRP3 and pro-IL-1β mRNA levels by inhibiting NF-κB/STAT3 phosphorylation	BV2 microglial cells, 5xFAD mice	Kim et al. (2021)

LRP3: nucleotide-binding oligomerization domain-like receptor pyrin domain-containing 3; AD: Alzheimer’s disease; Aβ: amyoid β; ATP: Adenosine triphosphate; P2X7: P2X purinergic receptor 7; APP: amyloid precursor protein; APP/PS1: APPswe/PS1dE9; SAMP8: senescence-accelerated mouse prone 8; NACHT: nucleotide-binding and oligomerization domain; LRR: leucine-rich repeat; NEK7: NIMA-related kinase 7; MNS: 3,4-methylenedioxy-β-nitrostyrene; BHB: *ß*-hydroxybutyric acid; TXNIP: Thioredoxin interacting protein; TRX: Thioredoxin; SFN: Sulforaphane; GB: Ginkgolide; ABPPκ: *Achyranthes* bidentate polypeptide fraction *κ*; PK: Picrorhiza kurroa; DHM: Dihydromyricetin; NSAIDs: Nonsteroidal anti-inflammatory drugs; IND: Indomethacin; AMPK: Adenosine 5′-monophosphate (AMP)-activated protein kinase; PG: Progesterone; TSG: 2,3,5,4′-Tetrahydroxystilbene-2-O-β-D-glucoside; Qu: Quercetin; ASC: apoptosis-associated speck-like protein containing a CARD; mtROS: mitochondrial ROS; NF-κB: nuclear factor kappa B; STAT3: Signal transducer and activator of transcription 3

### 4.1 IL-1β Antibodies and IL-1R Antagonists

IL-1β is usually present in cells as the precursor form pro-IL-1β. Pro-IL-1β has no biological activity. Activated caspase-1 can cleave pro-IL-1β into mature IL-1β through enzyme cleavage. At present, the strategy of targeting IL-1β has certain application prospects, but it also has some limitations. So far, there are mainly three biologics targeting IL-1β that have been used in the treatment of various inflammatory diseases. A recombinant IL-1 receptor antagonist anakinra, an inducible receptor rilonacept that binds to IL-1α and IL-1β, and the other one is IL-1β neutralizing antibody canakinumab (Giancane et al., 2016). These biological agents have been widely used to treat inflammation-related diseases, including autoimmune diseases (Sota et al., 2021), recurrent pericarditis (Fava et al., 2021), idiopathic arthritis (Autmizguine et al., 2015), gout (Perez-Ruiz et al., 2014). However, the clinical trials of these biologics in AD are rarely reported. Long-term injection of IL-1R blocking antibody to 3xTg-AD mice can significantly reduce brain inflammation, ameliorate cognitive impairment, relieve tau pathology, and partially reduce the level of Aβ oligomers (Kitazawa et al., 2011). In addition, Qi et al. found that anakinra can improve synaptic plasticity defects in a rat model of AD amyloidosis and eliminate the inhibitory effect on long-term potentiation (Qi et al., 2018). A recent study showed that anakinra can also alleviate synaptic loss and cognitive impairment in AD (Batista et al., 2021). For the application of inhibitors targeting IL-1β in AD, the ability of these biologics to cross the BBB, the ability to penetrate the brain tissue, and the side effects of drugs should be considered. Furthermore, upon NLRP3 inflammasome activation, in addition to the secretion of IL-1β, it also produces IL-18 and pyroptosis. Therefore, it is difficult to completely inhibit the pathogenic effect of NLRP3 inflammasome after blocking IL-1β. Recently, some new targets that participate in the regulation of NLRP3 inflammasome have been identified, which provides a new approach for AD therapy.

### 4.2 Specific Inhibitors of the NLRP3 Inflammasome

The activation of NLRP3 inflammasome depends on the integrity of the structure and function of NLRP3 and the assembly of NLRP3 inflammasome. Therefore, the potential therapeutic targets for NLRP3 inflammasome mainly include the NACHT domain of NLRP3, ASC and caspase-1, as well as other sites that affect its assembly. Targeting the pharmacological effects of NLRP3 inflammasome may be the best way to treat AD. Here, we mainly summarize several inhibitors for NLRP3 inflammasome activation and their therapeutic targets.

### 4.3 NLRP3 Activation Inhibitors

#### 4.3.1 Glyburide

Glyburide is a sulphonylurea drug approved by the FDA, which treats type 2 diabetes by blocking the ATP-sensitive K^+^ channel in *ß* pancreatic cells. Lamkanfi et al. found that glyburide has anti-inflammatory effects in an early study. It is the first identified compound that can inhibit the activation of NLRP3 inflammasome and the secretion of IL-1β induced by PAMPs, DAMPs and crystals. However, it has no effect on the activation of NLRC4 or NLRP1. The targets of glyburide still need to be further clarified. They found that glyburide targeted the signal components downstream of the P2X7 receptor and might act upstream of NLRP3 to inhibit the activation of caspase-1 (Lamkanfi et al., 2009). There are few studies on the application of glyburide in neurodegenerative diseases. Therefore, its therapeutic value in AD is still unclear.

#### 4.3.2 JC124

JC124 is a specific small molecule inhibitor of NLRP3 inflammasome. In 2018, Fulp et al. developed the methylated analogue JC124 based on the sulfonamide analogue JC121 of glyburide. After oral administration, JC124 can penetrate through the BBB and enter the brain tissue. After JC124 treatment, APP/PS1 transgenic mice shows significant improvement in cognitive impairment (Fulp et al., 2018). In addition, Yin et al. demonstrated that JC-124 inhibits the lysis and activation of caspase-1 in CRND8 APP transgenic mice (TgCRND8) mice, and selectively restrains the formation of NLRP3 inflammasome, thereby effectively reducing Aβ deposition and microglia activation (Yin et al., 2018). After treatment with JC124 in the traumatic brain injury (TBI) model, it can significantly inhibit the activation of NLRP3 induced by injury, reduce the expression level of its downstream effector protein, and thus play a role in neuroprotection (Kuwar et al., 2019). A recent study has reported the preventive efficacy of JC124 in AD. They found that JC124 has the ability to inhibit neuronal inflammation, regulate the accumulation of Aβ and promote the alleviation of cognitive impairment. Moreover, improved synaptic plasticity and endogenous neurogenesis in the hippocampus are also observed (Kuwar et al., 2021). Therefore, JC124 is a new type of inhibitor targeting NLRP3 inflammasome, which can reduce the neuropathology of AD and improve cognitive function, thereby exhibiting neuroprotective effects.

#### 4.3.3 Oridonin

Oridonin (Ori) is the main bioactive component of the natural anti-inflammatory Chinese medicinal herb Rabdosia rubescens, and has been proven to be as a specific covalent inhibitor of NLRP3 inflammasome. Under the stimulation of NLRP3 agonists such as monosodium urate crystals (MSU), ATP or cytosolic LPS (cLPS), Ori treatment inhibits NLRP3 inflammation and reduces IL-1β release (He et al., 2018). Regarding the target of Ori, studies have reported that Ori can directly bind to the NACHT domain of NLRP3. Ori forms a covalent bond with the cysteine 279 of NLRP3 in NACHT domain to block the interaction between NLRP3 and NEK7, thereby inhibiting NLRP3 inflammasome assembly and activation (He et al., 2018). In the AD mouse model, Ori inhibits the activation of microglia induced by Aβ1-42, reduces the release of inflammatory cytokines, prevents the loss of synapses, and improves the cognitive impairment of AD mice (Sulei Wang et al., 2014; Wang et al., 2016). In addition, it has also been observed in TBI that Ori treatment can significantly reduce the expression of NLRP3 inflammasome components (NLRP3, ASC and caspase-1), and restrict the secretion of IL-1β and IL-18 (Yan et al., 2020a). In addition to being widely used to treat inflammatory diseases, Ori also has potential neuroprotective effects. Therefore, Ori can be applied as a possible drug for long-term treatment of AD.

#### 4.3.4 CY-09

CY-09 is a selective and direct NLRP3 inhibitor. In 2017, Jiang et al. confirmed the target of interaction between CY-09 and NLRP3. They found that CY-09 can directly bind to the Walker A motif of NLRP3, rather than NLRC4, NLRP1, NOD2, or RIG-1, which indicates the specificity of CY-09. CY-09 directly binds to the ATP binding motif of the NLRP3 NACHT domain to inhibit NLRP3 ATPase activity, thereby inhibiting the assembly and activation of NLRP3 inflammasome (Jiang et al., 2017). Based on the high specificity and good pharmacokinetic characteristics of CY-09 targeting NLRP3, it may become a new method for the treatment of diseases. Currently, CY-09 can be treated for inflammatory related diseases such as osteoarthritis (Zhang et al., 2021a), myocardial fibrosis (Gao et al., 2021a), hepatic steatosis (Wang et al., 2021), etc. However, the effect of CY-09 has not been reported in AD, and its application value should be explored as soon as possible.

#### 4.3.5 MCC950

MCC950 is a small molecule compound of diarylsulfonylurea. It is a potent and selective small molecule inhibitor of NLRP3, which can block the activation of canonical and non-canonical NLRP3 at nanomolar concentrations. MCC950 specifically inhibits NLRP3 but not AIM2, NLRC4 or NLRP1 activation. MCC950 reduces IL-1β production *in vivo* and attenuates the severity of experimental autoimmune encephalomyelitis (EAE) (Coll et al., 2015). With the study of MCC950 target, the researchers have found that MCC950 can also specifically bind to NLRP3. It directly interacts with the walker B motif in the NACHT domain of NLRP3, which blocks the activity of NLRP3 ATPase and loses the ability to hydrolyze ATP, thereby blocking NLRP3 oligomerization and formation (Coll et al., 2019). This is further supported by another study. They have found that MCC950 can modify the active conformation of NLRP3 and prevent NLRP3 oligomerization (Tapia-Abellan et al., 2019). MCC950 is an effective and selective NLRP3 inhibitor, which has a wide range of applications in inflammatory diseases. However, here we mainly discuss the therapeutic effect of MCC950 in cognitive dysfunction diseases. Dempsey et al. found that, in the APP/PS1 AD mouse model, MCC950 can inhibit the activation of NLRP3 inflammasome in microglia, prevent the release of IL-1β, and promote the phagocytosis of Aβ by microglia, which reduces the accumulation of Aβ and improves the cognitive function (Qi et al., 2018). In addition, MCC950 can also completely inhibit the immune response after activation of NLRP3 inflammasomes induced by fibrils and low molecular weight Aβ aggregates (Luciunaite et al., 2020). MCC950 attenuates the reactivity of microglia induced by Aβ1-42 oligomers, blocks the activation of NLRP3 inflammasome, and eliminates memory impairment (Fekete et al., 2019). These results indicate that MCC950 can reduce Aβ-induced pathological events and enhance cognitive function. Some studies have also found that MCC950 improves the damage of synaptic plasticity (Qi et al., 2018), inhibits the activation of IL-1β induced by tau aggregates, and prevents tau-mediated pathological changes (Stancu et al., 2019). Li et al. reported that the administration of MCC950 improves the spatial memory and brain histology of senescence-accelerated mouse prone 8 (SAMP8), and reduces the deposition of Aβ in the mouse brain (Li et al., 2020). MCC950 may be a promising compound for AD treatment, but this also requires more animal experiments and clinical drug observation trials for further evaluation. The improvement of drugs based on MCC950 can reduce its side effects and increase its neuroprotective efficacy and safety, which is also a potential strategy for the development of AD drugs.

#### 4.3.6 OLT1177

OLT1177, also known as Dapansutrile, is an active *ß*-sulfonyl nitrile compound. OLT1177 is initially identified as a drug for the treatment of arthritis and is currently undergoing a phase II clinical trial for the treatment of acute gouty arthritis (Kluck et al., 2020). OLT1177 is a potent, selective and orally active inhibitor of NLRP3 inflammasome. The effect of OLT1177 is similar to that of MCC950. It blocks the canonical and non-canonical activation of NLRP3 inflammasome, and directly binds to NLRP3 to inhibit its ATPase activity (Marchetti et al., 2018). *In vitro* experiments have shown that nanomolar concentration of OLT1177 can specifically inhibit the activation of NLRP3 inflammasome and reduce the release of IL-1β and IL-18 (Marchetti et al., 2018). In a study, Lonnemann et al. provided some convincing evidence. Their results showed that OLT1177 inhibits the activation of NLRP3 inflammasome, thereby improving cognitive dysfunction and synaptic plasticity in AD mice, reducing the number of pathological plaque deposits in the cerebral cortex, and reducing the activity of microglia (Lonnemann et al., 2020). However, there are still few researches on the application of OLT1177 in neurodegenerative diseases. In short, considering that OLT1177 has good safety, pharmacokinetics and less side effects after oral administration, this makes OLT1177 to become an option for the treatment of AD in the future.

#### 4.3.7 Tranilast

Tranilast is originally used as an anti-allergic drug, which has a good therapeutic effect on asthma, allergic rhinitis, idiopathic dermatitis and other allergic diseases. Now, other uses, such as myocardial fibrosis and anti-cancer treatment, are gradually being discovered (Chen et al., 2021; Osman et al., 2021). In 2018, Huang et al. first discovered that Tranilast is a direct NLRP3 inhibitor that can inhibit the NLRP3-NLRP3 interaction. Tranilast inhibits NLRP3 inflammasome activation in macrophages, but has no effects on AIM2 or NLRC4 inflammasome activation. Tranilast directly binds to the NACHT domain of NLRP3 and suppresses the assembly of NLRP3 inflammasome by blocking NLRP3 oligomerization (Huang et al., 2018). Moreover, the researchers have also reported that Tranilast increases the lysine 63 (K63)-linked ubiquitination of NLRP3, restricts NLRP3 oligomerization, blocks the assembly and activation of NLRP3 inflammasome, thereby improving vascular inflammation and atherosclerosis in Ldlr^−/-^ and ApoE^−/-^ mice (Chen et al., 2020). Tranilast can inhibit the formation of rat gliomas after oral administration, which indicates that Tranilast can cross the BBB (Platten et al., 2001). However, the therapeutic effect of Tranilast in AD is still unclear. Recent studies have reported that Tranilast can improve cognitive behavioral parameters and significantly increase memory-related proteins in Aβ-induced cognitive deficit model mice, thereby showing the potential for neuroprotection (Thapak et al., 2021). On the contrary, some researchers have put forward different views. Connors et al. found that Tranilast is likely to promote fibrillation by shifting Aβ monomer conformations to those capable of seed formation and fibril elongation, which indicates that elderly patients treated with Tranilast may increase the risk of AD (Connors et al., 2013). The role of Tranilast in AD still needs further research, and whether Tranilast plays a role in AD by inhibiting the activation of NLRP3 inflammasome is also unknown.

#### 4.3.8 BAY 11–7082 and Parthenolide

BAY 11–7082 and Parthenolide are common NF-κB inhibitors. BAY 11–7082 can inhibit IκBα phosphorylation and prevent nuclear translocation of NF-κB. Parthenolide is a powerful natural anti-inflammatory drug derived from the medicinal plant Feverfew. As early as 2010, Juliana et al. found that BAY 11–7082 and Parthenolide can selectively inhibit the activity of NLRP3 inflammasome in macrophages, but this effect is not related to their inhibitory effect on NF-κB activity. They found that Bay 11–7082 and Parthenolide blocks ASC oligomerization via inhibiting NLRP3 ATPase activity. Surprisingly, in addition to directly inhibiting NLRP3, Parthenolide is also a direct inhibitor of caspase-1, while Bay 11–7082 has no such effect. Therefore, Bay 11–7082 selectively inhibits the NLRP3 inflammasome pathway, while Parthenolide inhibits the activity of multiple inflammasome pathways (Juliana et al., 2010). In the TBI model, Bay 11–7082 shows a similar effect to NLRP3 knockout, which significantly limits the NLRP3 inflammasome activation, reduces the levels of caspase-1 and IL-1β, and improves the cognitive function of model mice (Irrera et al., 2017). Additionally, the pretreatment of Bay 11–7082 can also block the activation of inflammasome through the pharmacological inhibition of NF-κB/NLRP3, thereby reducing neuronal damage and cognitive dysfunction in aged rats (Liu et al., 2021). In APP23 mice treated with kainic acid (KA), BAY 11–7082 attenuates KA-induced neuronal degeneration and Aβ deposition by inhibiting the activation of NLPR3 inflammasome, and ultimately improves the cognitive function (Ruan et al., 2019). These studies indicate that BAY 11–7082 has neuroprotective effects on AD. Parthenolide has been proven to have antioxidant and anti-inflammatory effects, but its role in the nervous system has not yet been elucidated. According to reports, Parthenolide can effectively reduce neuroinflammation and improve brain damage (Jun-An Wang et al., 2020). More importantly, the synthesis of Parthenolide derivatives with low toxicity, such as compound 8b (Ou et al., 2020), may bring hope for targeting NLRP3 inflammasome to treat AD.

#### 4.3.9 3,4-Methylenedioxy-β-nitrostyrene

3,4-methylenedioxy-β-nitrostyrene (MNS) is a tyrosine kinase inhibitor. In 2014, He et al. first discovered the inhibitory effect of MNS on NLRP3 inflammasome activation. They found that MNS do not affect the activation of NLRC4 or AIM2 inflammasome, but specifically blocks NLRP3-mediated ASC speck formation and oligomerization. MNS directly binds to the nucleotide-binding and oligomerization domain (NOD) and leucine-rich repeat (LRR) domains of NLRP3 and inhibits the activity of NLRP3 ATPase, thereby blocking the assembly and activation of inflammasome (He et al., 2014). It has previously been reported that MNS can inhibit platelet aggregation, tumor cell invasion and metastasis (Wang et al., 2007; Chen et al., 2015). At present, more attention should be paid to the application of MNS in inflammatory-related diseases by blocking the activation of NLRP3 inflammasome. The role of MNS in AD is still unknown, which requires more *in vivo* and *in vitro* experiments.

### 4.4 ASC Oligomerization Inhibitors

#### 4.4.1 β-hydroxybutyrate

β-hydroxybutyric acid (BHB) is a ketone body produced by the oxidation of fatty acids in the liver under fasting conditions, which can provide alternative energy for the brain and heart. In 2015, Youm et al. first discovered that BHB can specifically inhibit the activation of NLRP3 inflammasome. They found that BHB inhibits NLRP3 inflammasome assembly and activation by preventing K^+^ efflux and reducing ASC oligomerization and speck formation (Youm et al., 2015). Clinical evidence shows that long-term consumption of ketogenic formula can significantly improve the cognitive function of AD patients (Ota et al., 2019). In addition, BHB reduces the level of IL-1β by inhibiting NLRP3-mediated hippocampal neuroinflammation, thereby exerting an antidepressant effect (Yamanashi et al., 2017; Kajitani et al., 2020). Subsequently, BHB is also found to attenuate long-term stress-induced anxiety-related behaviors and plays an anti-anxiety effect (Yamanashi et al., 2020). Recently, in the 5xFAD mouse model, Shippy et al. revealed that the administration of BHB reduces Aβ plaque formation, microglial proliferation, ASC formation and caspase-1 activation, thereby alleviating AD pathology (Shippy et al., 2020). BHB can easily cross the BBB, which increases its therapeutic potential as a treatment strategy for AD.

### 4.5 Caspase-1 Activation Inhibitors

#### 4.5.1 VX-765

Caspase-1 is an important component of NLRP3 inflammasome. Upon activation, caspase-1 promotes the production of IL-1β/IL-18, and at the same time mediates the pyroptosis through gasdermin D. VX-765 is a safe, effective, selective, and small molecule caspase-1 inhibitor. Early studies showed that VX-765 inhibits the production of IL-1β in forebrain astrocytes, thereby blocking epilepsy in rats (Ravizza et al., 2008). Currently, VX-765 has entered phase II clinical trials for patients with epilepsy. VX-765 is a non-toxic caspase-1 inhibitor that is permeable to the BBB. In the AD model, VX-765 prevents progressive Aβ deposition and reverses brain inflammation, synaptic loss, and memory impairment (Flores et al., 2018). In addition, VX-765 is promising as an effective drug to prevent the onset of cognitive deficits. Research by Flores et al. showed that treatment with VX-765 for 1 month before the onset of symptoms in AD J20 model mice could delay the cognitive impairment of mice by at least 5 months (Flores et al., 2020). Therefore, VX-765 represents a safe drug, which may have potential value in the early prevention of AD cognitive deficits and the improvement of cognitive dysfunction.

#### 4.5.2 Ac-YVAD-CMK

Ac-YVAD-CMK is a selective and irreversible inhibitor of caspase-1, and prevents the expression of IL-1β. Ac-YVAD-CMK can inhibit the activation and infiltration of microglia around the hematoma in the rat model of cerebral hemorrhage, promote the transformation of microglia from M1 type to M2 type, and reduce the release of IL-1β/IL-18. At the same time, Ac-YVAD-CMK inhibits cell pyroptosis, improves nerve function, and exhibits neuroprotective effect (Lin et al., 2018; Liang et al., 2019). Infusion of Ac-YVAD-CMK into the lateral ventricle of aged rats can inhibit the production of hippocampal IL-1β, thereby improving the memory of aged rats and reversing the decrease of hippocampal neurons (Gemma et al., 2005, Gemma et al., 2007). In the AD model, AC-YVAD-CMK treatment improves spatial learning and memory impairment in APP/PS1 mice, reduces Aβ plaque deposition, and promotes membrane transport of GluA1 (Gu et al., 2021).

### 4.6 Plant-Derived Compounds and Chinese Herbal Medicines

Some plant-derived compounds and Chinese herbal medicines can inhibit the activation of NLRP3 inflammasome, and exhibit the effect of preventing and treating AD. Resveratrol is a natural polyphenol compound extracted from plants. Many studies have shown that resveratrol has anti-cancer, anti-oxidant, anti-inflammatory, anti-aging and other pharmacological effects (Kumar et al., 2021). In the nervous system, resveratrol can play a neuroprotective effect by inhibiting the activation of NLRP3 inflammasome. Feng et al. found that resveratrol significantly inhibits the proliferation and activation of BV-2 cells induced by Aβ through the TXNIP/TRX/NLRP3 signaling pathway, and reduces the expression levels of caspase-1 and IL-1β (Feng and Zhang 2019). Qi et al. also reported that resveratrol reduces Aβ-induced IL-1β production and mitochondrial dysfunction through the NF-κB/IL-1β/NLRP3 signaling pathway, improves learning and cognitive impairment, and plays an anti-dementia effect (Qi et al., 2019). Picrorhiza kurroa (PK) is a herbal medicine with antioxidant, anti-inflammatory, anti-allergic and anti-cancer effects. Kim et al. found that, in the hippocampus of 5xFAD mice, PK inhibits the activation of NLRP3 inflammasome, reduces the protein expression level of NLRP3 and the activity of caspase-1, thereby blocking the release of IL-1β (Kim et al., 2020). Dihydromyricetin (DHM) is a kind of plant flavonoids, which has many unique effects such as anti-oxidation, anti-thrombosis, anti-cancer, and anti-alcoholism. It is convinced that flavonoids can cross the BBB to regulate inflammation and exert neuroprotective effects (Youdim et al., 2003). In the AD model, DHM treatment can inhibit the activation of NLRP3 inflammasome in APP/PS1 mice and reduce the level of IL-1β. DHM, as a therapeutic drug that inhibits the activation of microglia by inhibiting NLRP3 inflammasome, contributes to prevent the progression of AD-like pathology and improve spatial memory (Feng et al., 2018). Pterostilbene is a natural compound with antioxidant, anti-inflammatory and neuroprotective activities. Li et al. reported that pterostilbene attenuates the neuroinflammatory response induced by Aβ1-42 in microglia via inhibiting the NLRP3/caspase-1 inflammasome pathway (Li et al., 2018b). Sulforaphane (SFN) is an isothiocyanate derivative contained in cruciferous vegetables. SFN’s anti-oxidation, anti-cancer, anti-inflammatory and other uses are being extensively studied. SFN also exhibits anti-inflammatory effects in the brain. Tufekci et al. found that SFN inhibits the secretion of IL-1β and IL-18 mediated by NLRP3 inflammasomes and the pyroptosis of microglia (Tufekci et al., 2021). Ginkgolide B (GB) is a plant ester compound extracted from Ginkgo biloba leaves. Through its anti-inflammatory, anti-oxidant and anti-apoptotic properties, GB exerts an effective neuroprotective effect on ischemic brain injury and neurodegenerative diseases. Zhang et al. found that GB treatment prevents the pathological process of AD and inhibits neuroinflammation by inhibiting NLRP3 inflammasome activation and promoting microglia M2 polarization (Zhang et al., 2021). *Achyranthes* bidentate has anti-inflammatory and antioxidant activities, and has been used in traditional Chinese medicine for the treatment of dementia and osteoporosis for a long time. Recent study has shown that *Achyranthes* bidentate polypeptide fraction κ (ABPPκ) can down-regulate Aβ oligomer-induced IκBα phosphorylation and NLRP3 expression *in vitro*. *In vivo*, pre-administration of ABPPk inhibits the activation of microglia in the CA3 region of the hippocampus, promotes the polarization of the microglia M2 phenotype, and reduces the expression of NLRP3, cleaved caspase-1 and ASC in the brain, thereby significantly improving the cognitive impairment of mice (Ge et al., 2021). In addition, there are other Chinese herbal medicines, including Dl-3-n-butylphthalide (Dl-NBP) (Wang et al., 2019), Shaoyao Gancao Tang (SG-Tang) (Chiu et al., 2021), and Liquiritigenin (LG) (Du et al., 2021), which can play a neuroprotective role in AD by inhibiting the NLRP3 inflammasome pathway. Therefore, these Chinese herbal medicines and extracts that can inhibit the activation of NLRP3 may be a promising and safe treatment for AD.

### 4.7 Nonsteroidal Anti-inflammatory Drugs

Nonsteroidal anti-inflammatory drugs (NSAIDs) are the most widely used anti-inflammatory drugs in clinical practice. These drugs have a wide range of effects, including anti-inflammatory, anti-rheumatic, antipyretic, analgesic and so on, which are widely applied in rheumatic and painful diseases. NSAIDs mainly act by inhibiting the cyclooxygenase-1 (COX-1) and COX-2. Researches have shown that NSAIDs can delay the development or reduce the risk of AD by acting on the NLRP3 inflammasome pathway (Deardorff and Grossberg 2017). Early studies found that NSAIDs of the fenamate class (such as mefenamic acid) are effective and selective inhibitors of NLRP3 inflammasome, which can selectively inhibit the activation of NLRP3 inflammasome in macrophages and relieve the cognitive impairment of AD mice. The effect of NSAIDs on NLRP3 may be through the inhibition of the volume-regulated anion channel (VRAC), independently of COX enzymes (Daniels et al., 2016). Another study showed that Indomethacin (IND) reduces the expression of IL-1β and caspase-1 via inhibiting NLRC4 and NLRP3 inflammasomes, thereby improving neuroinflammation and memory impairment in AD (Karkhah et al., 2021). In a recent review, Hampel et al. have reported that, in transgenic AD mice, the researchers have found that NSAIDs not only exert neuroprotective effects by suppressing inflammatory effects, but also reduce early Aβ pathology through other mechanisms, thereby preventing memory decline. However, all controlled prospective trials from the clinic have not found positive therapeutic effects of NSAIDs in AD patients, or have limited their application due to the severe side effects (Hampel et al., 2020). So the researchers do not have positive data from patients supporting the hypothesis that NSAIDs are effective in AD. In humans, the occurrence of AD is related to many predisposing factors (such as age, genetics and environment, etc.), and its pathogenic mechanisms are also complex and diverse. This may lead to different mechanistic pathways for human AD and rodent AD disease models. Furthermore, human AD is a long-term asymptomatic chronic disease, and the relatively late treatment time point may also be a potential reason for the clinical inefficiency of NSAIDs. Because epidemiological data show that the incidence of AD decreases after long-term treatment with NSAIDs (Hampel et al., 2020). In the future, evaluation of the effect of NSAIDs in AD treatment requires more data from clinical trials.

### 4.8 MicroRNAs

MicroRNAs can directly target the NLRP3 inflammasome, and play an important role in the regulation of inflammation. MiR-138–5p can directly target the 3′-UTR of NLRP3 and inhibit the expression of NLRP3. Up-regulation of miR-138–5p inhibits the activation of NLRP3/caspase-1 axis and microglia, thereby attenuating hippocampal neuroinflammation and improving the cognitive function of the rat model (Feng et al., 2021a). MicroRNA-223 also directly targets and inhibits the expression of NLRP3, thereby reducing LPS-induced inflammation in microglia and improving neuronal function (Zhang et al., 2020). However, some studies have found that microRNAs also affect the expression of NLRP3 indirectly. MiR-194–5p can target TNF receptor associated factor 6 (TRAF6), which interacts with NLRP3 to promote the activation of NLRP3 inflammasome. Overexpression of miR-194–5p can reduce the interaction of TRAF6/NLRP3, thereby inhibiting the NLRP3 inflammasome activation and reducing neuroinflammation (Wan et al., 2021). A recent study found that the expression level of miR-22 in the peripheral blood of AD patients is lower than that of healthy people. MiR-22 regulates glial cell pyroptosis by targeting GSDMD, inhibits the activation of NLRP3 inflammasome, and reduces the release of inflammatory cytokines, thereby alleviating cognitive impairment in AD mice (Han et al., 2020a). In addition, miR-34c (Xu et al., 2019), miR-30e (Li et al., 2018c), and miR-7 (Zhou et al., 2016) can also directly target and inhibit NLRP3, regulate the activity of NLRP3 inflammasome, and improve the occurrence of neuroinflammation. Perhaps targeting microRNAs for regulating the activation of NLRP3 inflammasome may be a new direction for AD treatment.

### 4.9 Autophagy and Mitophagy Activators

As described in the previous regulation of NLRP3 inflammasome activation, autophagy and mitophagy have been shown to regulate inflammasome activation. Therefore, any drugs that activate autophagy or mitophagy can negatively regulate NLRP3 inflammasome. IIIM-941 can induce autophagy through AMPK pathway to inhibit ATP-induced NLRP3 inflammasome activity (Ali et al., 2021). Dopamine D1 receptor agonist A-68930 enhances the degradation of NLRP3 inflammasome and reduces the secretion of IL-1β and IL-18 by activating the AMPK/autophagy signaling pathway, thereby improving the neuroinflammation and cognitive impairment of mice induced by Aβ1-42 (Cheng et al., 2020). Progesterone (PG) is a steroid with neuroprotective effects. Hong et al. found that PG inhibits the activation of NLRP3-caspase-1 inflammasome induced by Aβ via enhancing the autophagy of astrocytes, thereby exhibiting neuroprotective effects (Hong et al., 2019). In the TgCRND8 AD model, Ginkgo biloba extract EGb 761 can activate autophagy in microglia, down-regulate the level of NLRP3 protein, reduce the activation of IL-1β and caspase-1 induced by Aβ, and improve the cognitive ability of mice (Liu et al., 2015). Moreover, AICAR and metformin can activate PRKAA1 to enhance autophagy, which not only contributes to clear extracellular Aβ fibrils, but also inhibits Aβ-induced NLRP3 inflammasome activation and IL-1β release (Cho et al., 2014). 2,3,5,4′-Tetrahydroxystilbene-2-O-β-D-glucoside (TSG) is one of the main active ingredients extracted from Polygonum multiflorum. It has been proven to be used in the treatment of AD. TSG can effectively alleviate the neuroinflammatory response induced by LPS via inhibiting the NLRP3 signaling pathway in microglia and neurons. In addition, TSG can also prevent LPS/ATP and Aβ-induced inflammation through AMPK/PINK1/Parkin-dependent enhancement of mitophagy, thereby exerting a neuroprotective effect (Gao et al., 2020). Quercetin (Qu) is a natural flavonoid compound with anti-inflammatory and antioxidant properties. Recent studies have found that Qu promotes mitophagy to enhance the clearance of damaged mitochondria, thereby inhibiting mtROS-mediated activation of NLRP3 inflammasome in microglia, and preventing neuronal damage (Han et al., 2021). We believe that the enhancement of autophagy and mitophagy in microglia may be a new strategy for the treatment of AD, but the safety of this treatment remains to be further observed.

## 5 ROS and NF-κB Inhibitors

α-lipoic acid (LA) is an antioxidant, and is frequently used in the treatment of diabetes. LA can easily pass through the BBB and play a protective role in the nervous system. Studies found that *α*-LA inhibits the activation of NLRP3 inflammasome in microglia, regulates the polarization of microglia from the M1 phenotype to the M2 phenotype, and reduces the neuroinflammatory response (Kim et al., 2019). Edaravone (EDA) is a free radical scavenger that has neuroprotective effects on cerebral infarction, amyotrophic lateral sclerosis and dementia. In the AD cell model, EDA significantly attenuates mitochondrial membrane potential (∆ψm), reduces the production of mtROS, and inhibits the activation of NLRP3 inflammasome induced by Aβ (Wang et al., 2017). Donepezil is a reversible central acetylcholinesterase (AChE) inhibitor that can be used to improve the cognitive function of AD patients. Recent studies found that Donepezil can also inhibit LPS-induced AKT/MAPK signaling and NF-κB/STAT3 phosphorylation in BV2 microglia, and down-regulate NLRP3 and pro-IL-1β mRNA levels, thereby reducing neuroinflammation induced by NLRP3 inflammasome (Kim et al., 2021).

## 6 Conclusion and Future Perspectives

It has been nearly 2 decades since the NLRP3 inflammasome being discovered. With continuous studies, researchers have gained a certain understanding of the structure, composition, regulation and role of NLRP3, but its precise molecular mechanisms in diseases have not been fully elucidated. In recent years, the research of NLRP3 inflammasome in neurodegenerative diseases has attracted much attention. More and more evidences have confirmed that NLRP3 inflammasome activation plays an important role in the pathogenesis and progression of AD. More importantly, microglia and astrocytes play a crucial role in the chronic neuroinflammatory response of AD caused by NLRP3 inflammasome. In AD cells and animal models, the inhibitory measures against NLRP3 or its inflammasome constituent molecules can alleviate the inflammatory response, and reduce Aβ deposition, Tau phosphorylation and other pathological features, thereby improving AD-related behavioral abnormalities. Therefore, targeting NLRP3 inflammasome may be a new trend for AD treatment. The activation of NLRP3 inflammasome involves upstream signal related regulatory factors, priming signal, activation signal and downstream IL-1β and IL-18 effectors. In the early stage of drug development, researchers usually focus on strategies to block downstream inflammatory cytokines. Inhibitors targeting IL-1β as drugs for the treatment of neurological diseases have not achieved satisfactory clinical results. With the discovery of new drug targets, people gradually turn their attentions to NLRP3 and the constituent molecules ASC and caspase-1. This targeting effect is selective and efficient, which can ensure the specificity of the treatment to the greatest extent and reduce non-specific effects. In addition, the upstream-related regulatory factors of NLRP3 inflammasome activation can also become attractive pharmacological targets, but due to the complexity of the interaction of upstream signals, it may bring non-specific therapeutic roles. So far, although many compounds have successfully been identified to target NLRP3 inflammasome *in vitro* and *in vivo*, their therapeutic effects and safety in AD patients have yet to be verified by clinical trials. In the CNS diseases, the development of therapeutic drugs targeting the NLRP3 inflammasome needs to be evaluated by its permeability across the BBB. More importantly, under the premise of obtaining the desired therapeutic values, it will not cause toxic effects on the whole-body or CNS. In addition, AD is a long-term chronic progressive disease, and usually requires intervention in the early stage of the disease. However, whether long-term use of targeted drugs for inflammasomes will affect the health of AD patients requires further evaluation. In view of the good safety and side effects of traditional Chinese herbal medicines and plant-derived compounds, they may provide new directions for the treatment of AD.

## References

[B1] Ahmed M. E. Iyer S. Thangavel R. Kempuraj D. Selvakumar G. P. Raikwar S. P. (2017). Co-Localization of Glia Maturation Factor with NLRP3 Inflammasome and Autophagosome Markers in Human Alzheimer's Disease Brain. J. Alzheimers Dis. 60, 1143–1160. 10.3233/JAD-170634 28984607 PMC5770146

[B2] Ali M. Gupta M. Wani A. Sharma A. Abdullaha M. Kour D. (2021). IIIM-941, a Stilbene Derivative Inhibits NLRP3 Inflammasome Activation by Inducing Autophagy. Front. Pharmacol. 12, 695712. 10.3389/fphar.2021.695712 34248643 PMC8267097

[B3] Allen I. C. Scull M. A. Moore C. B. Holl E. K. McElvania-TeKippe E. Taxman D. J. (2009). The NLRP3 Inflammasome Mediates *In Vivo* Innate Immunity to Influenza A Virus through Recognition of Viral RNA. Immunity 30, 556–565. 10.1016/j.immuni.2009.02.005 19362020 PMC2803103

[B4] Amores-Iniesta J. Barberà-Cremades M. Martínez C. M. Pons J. A. Revilla-Nuin B. Martínez-Alarcón L. (2017). Extracellular ATP Activates the NLRP3 Inflammasome and Is an Early Danger Signal of Skin Allograft Rejection. Cell Rep 21, 3414–3426. 10.1016/j.celrep.2017.11.079 29262323 PMC5746605

[B5] Apelt J. Schliebs R. (2001). Beta-amyloid-induced Glial Expression of Both Pro- and Anti-inflammatory Cytokines in Cerebral Cortex of Aged Transgenic Tg2576 Mice with Alzheimer Plaque Pathology. Brain Res. 894, 21–30. 10.1016/s0006-8993(00)03176-0 11245811

[B6] Autmizguine J. Cohen-Wolkowiez M. Ilowite N. (2015). Rilonacept Pharmacokinetics in Children with Systemic Juvenile Idiopathic Arthritis. J. Clin. Pharmacol. 55, 39–44. 10.1002/jcph.372 25079592 PMC4276471

[B7] Bai H. Yang B. Yu W. Xiao Y. Yu D. Zhang Q. (2018). Cathepsin B Links Oxidative Stress to the Activation of NLRP3 Inflammasome. Exp. Cel Res. 362, 180–187. 10.1016/j.yexcr.2017.11.015 29196167

[B8] Batista A. F. Rody T. Forny-Germano L. Cerdeiro S. Bellio M. Ferreira S. T. (2021). Interleukin-1β Mediates Alterations in Mitochondrial Fusion/fission Proteins and Memory Impairment Induced by Amyloid-β Oligomers. J. Neuroinflammation 18, 54. 10.1186/s12974-021-02099-x 33612100 PMC7897381

[B9] Bauernfeind F. Bartok E. Rieger A. Franchi L. Núñez G. Hornung V. (2011). Cutting Edge: Reactive Oxygen Species Inhibitors Block Priming, but Not Activation, of the NLRP3 Inflammasome. J. Immunol. 187, 613–617. 10.4049/jimmunol.1100613 21677136 PMC3131480

[B10] Bauernfeind F. G. Horvath G. Stutz A. Alnemri E. S. MacDonald K. Speert D. (2009). Cutting Edge: NF-kappaB Activating Pattern Recognition and Cytokine Receptors License NLRP3 Inflammasome Activation by Regulating NLRP3 Expression. J. Immunol. 183, 787–791. 10.4049/jimmunol.0901363 19570822 PMC2824855

[B11] Benilova I. Karran E. De Strooper B. (2012). The Toxic Aβ Oligomer and Alzheimer's Disease: an Emperor in Need of Clothes. Nat. Neurosci. 15, 349–357. 10.1038/nn.3028 22286176

[B12] Byeon H. E. Jeon J. Y. Kim H. J. Kim D. J. Lee K. W. Kang Y. (2017). MicroRNA-132 Negatively Regulates Palmitate-Induced NLRP3 Inflammasome Activation through FOXO3 Down-Regulation in THP-1 Cells. Nutrients 9, 1370. 10.3390/nu9121370 PMC5748820 29258239

[B13] Cardoso S. M. Santana I. Swerdlow R. H. Oliveira C. R. (2004). Mitochondria Dysfunction of Alzheimer's Disease Cybrids Enhances Abeta Toxicity. J. Neurochem. 89, 1417–1426. 10.1111/j.1471-4159.2004.02438.x 15189344

[B14] Cha M. Y. Han S. H. Son S. M. Hong H. S. Choi Y. J. Byun J. (2012). Mitochondria-specific Accumulation of Amyloid β Induces Mitochondrial Dysfunction Leading to Apoptotic Cell Death. PLoS ONE 7, e34929. 10.1371/journal.pone.0034929 22514691 PMC3325919

[B15] Chen I. H. Chang F. R. Wu Y. C. Kung P. H. Wu C. C. (2015). 3,4-Methylenedioxy-β-nitrostyrene Inhibits Adhesion and Migration of Human Triple-Negative Breast Cancer Cells by Suppressing β1 Integrin Function and Surface Protein Disulfide Isomerase. Biochimie 110, 81–92. 10.1016/j.biochi.2015.01.006 25593085

[B16] Chen S. Wang Y. Pan Y. Liu Y. Zheng S. Ding K. (2020). Novel Role for Tranilast in Regulating NLRP3 Ubiquitination, Vascular Inflammation, and Atherosclerosis. J. Am. Heart Assoc. 9, e015513. 10.1161/JAHA.119.015513 32476536 PMC7429049

[B17] Chen Y. Huang M. Yan Y. He D. (2021). Tranilast Inhibits Angiotensin II-Induced Myocardial Fibrosis through S100A11/Transforming Growth Factor-β (TGF-β1)/Smad axis. Bioengineered 12, 8447–8456. 10.1080/21655979.2021.1982322 34663163 PMC8806955

[B18] Cheng Z. Y. Xia Q. P. Hu Y. H. Wang C. He L. (2020). Dopamine D1 Receptor Agonist A-68930 Ameliorates Aβ1-42-Induced Cognitive Impairment and Neuroinflammation in Mice. Int. Immunopharmacol. 88, 106963. 10.1016/j.intimp.2020.106963 33182028

[B19] Chiu Y. J. Lin C. H. Lee M. C. Hsieh-Li H. M. Chen C. M. Wu Y. R. (2021). Formulated Chinese Medicine Shaoyao Gancao Tang Reduces NLRP1 and NLRP3 in Alzheimer's Disease Cell and Mouse Models for Neuroprotection and Cognitive Improvement. Aging (Albany NY) 13, 15620–15637. 10.18632/aging.203125 34106880 PMC8221334

[B20] Cho M. H. Cho K. Kang H. J. Jeon E. Y. Kim H. S. Kwon H. J. (2014). Autophagy in Microglia Degrades Extracellular β-amyloid Fibrils and Regulates the NLRP3 Inflammasome. Autophagy 10, 1761–1775. 10.4161/auto.29647 25126727 PMC4198361

[B21] Coll R. C. Hill J. R. Day C. J. Zamoshnikova A. Boucher D. Massey N. L. (2019). MCC950 Directly Targets the NLRP3 ATP-Hydrolysis Motif for Inflammasome Inhibition. Nat. Chem. Biol. 15, 556–559. 10.1038/s41589-019-0277-7 31086327

[B22] Coll R. C. Robertson A. A. Chae J. J. Higgins S. C. Muñoz-Planillo R. Inserra M. C. (2015). A Small-Molecule Inhibitor of the NLRP3 Inflammasome for the Treatment of Inflammatory Diseases. Nat. Med. 21, 248–255. 10.1038/nm.3806 25686105 PMC4392179

[B23] Connors C. R. Rosenman D. J. Lopes D. H. Mittal S. Bitan G. Sorci M. (2013). Tranilast Binds to Aβ Monomers and Promotes Aβ Fibrillation. Biochemistry 52, 3995–4002. 10.1021/bi400426t 23679559 PMC4082028

[B24] d'Errico P. Meyer-Luehmann M. (2020). Mechanisms of Pathogenic Tau and Aβ Protein Spreading in Alzheimer's Disease. Front. Aging Neurosci. 12, 265. 10.3389/fnagi.2020.00265 33061903 PMC7481386

[B25] Daniels M. J. Rivers-Auty J. Schilling T. Spencer N. G. Watremez W. Fasolino V. (2016). Fenamate NSAIDs Inhibit the NLRP3 Inflammasome and Protect against Alzheimer's Disease in Rodent Models. Nat. Commun. 7, 12504. 10.1038/ncomms12504 27509875 PMC4987536

[B26] de Brito O. M. Scorrano L. (2008). Mitofusin 2 Tethers Endoplasmic Reticulum to Mitochondria. Nature 456, 605–610. 10.1038/nature07534 19052620

[B27] Deardorff W. J. Grossberg G. T. (2017). Targeting Neuroinflammation in Alzheimer's Disease: Evidence for NSAIDs and Novel Therapeutics. Expert Rev. Neurother 17, 17–32. 10.1080/14737175.2016.1200972 27293026

[B28] Dela Cruz C. S. Kang M. J. (2018). Mitochondrial Dysfunction and Damage Associated Molecular Patterns (DAMPs) in Chronic Inflammatory Diseases. Mitochondrion 41, 37–44. 10.1016/j.mito.2017.12.001 29221810 PMC5988941

[B29] Dempsey C. Rubio Araiz A. Bryson K. J. Finucane O. Larkin C. Mills E. L. (2017). Inhibiting the NLRP3 Inflammasome with MCC950 Promotes Non-phlogistic Clearance of Amyloid-β and Cognitive Function in APP/PS1 Mice. Brain Behav. Immun. 61, 306–316. 10.1016/j.bbi.2016.12.014 28003153

[B30] Ding W. Liu T. Bi X. Zhang Z. (2017). Mitochondria-Targeted Antioxidant Mito-Tempo Protects against Aldosterone-Induced Renal Injury *In Vivo* . Cell. Physiol. Biochem. 44, 741–750. 10.1159/000485287 29169180

[B31] Downs K. P. Nguyen H. Dorfleutner A. Stehlik C. (2020). An Overview of the Non-canonical Inflammasome. Mol. Aspects Med. 76, 100924. 10.1016/j.mam.2020.100924 33187725 PMC7808250

[B32] Du Y. Luo M. Du Y. Xu M. Yao Q. Wang K. (2021). Liquiritigenin Decreases Aβ Levels and Ameliorates Cognitive Decline by Regulating Microglia M1/M2 Transformation in AD Mice. Neurotox Res. 39, 349–358. 10.1007/s12640-020-00284-z 32990912

[B33] Duan Y. Kelley N. He Y. (2020). Role of the NLRP3 Inflammasome in Neurodegenerative Diseases and Therapeutic Implications. Neural Regen. Res. 15, 1249–1250. 10.4103/1673-5374.272576 31960806 PMC7047811

[B34] Dunn A. R. O'Connell K. M. S. Kaczorowski C. C. (2019). Gene-by-environment Interactions in Alzheimer's Disease and Parkinson's Disease. Neurosci. Biobehav Rev. 103, 73–80. 10.1016/j.neubiorev.2019.06.018 31207254 PMC6700747

[B35] Fava A. M. Reyaldeen R. Lo Presti S. Goyal A. Akintoye E. Hughes D. (2021). Rilonacept for the Treatment of Recurrent Pericarditis. Expert Opin. Biol. Ther. 31, 1–10. 10.1080/14712598.2022.2005024 34757872

[B36] Fekete C. Vastagh C. Dénes Á. Hrabovszky E. Nyiri G. Kalló I. (2019). Chronic Amyloid β Oligomer Infusion Evokes Sustained Inflammation and Microglial Changes in the Rat Hippocampus via NLRP3. Neuroscience 405, 35–46. 10.1016/j.neuroscience.2018.02.046 29522854

[B37] Feng J. Wang J. X. Du Y. H. Liu Y. Zhang W. Chen J. F. (2018). Dihydromyricetin Inhibits Microglial Activation and Neuroinflammation by Suppressing NLRP3 Inflammasome Activation in APP/PS1 Transgenic Mice. CNS Neurosci. Ther. 24, 1207–1218. 10.1111/cns.12983 29869390 PMC6282966

[B38] Feng L. Zhang L. (2019). Resveratrol Suppresses Aβ-Induced Microglial Activation through the TXNIP/TRX/NLRP3 Signaling Pathway. DNA Cel Biol 38, 874–879. 10.1089/dna.2018.4308 31215797

[B39] Feng X. Hu J. Zhan F. Luo D. Hua F. Xu G. (2021a). MicroRNA-138-5p Regulates Hippocampal Neuroinflammation and Cognitive Impairment by NLRP3/Caspase-1 Signaling Pathway in Rats. J. Inflamm. Res. 14, 1125–1143. 10.2147/JIR.S304461 33814920 PMC8009546

[B40] Feng Y. S. Tan Z. X. Wu L. Y. Dong F. Zhang F. (2021b). The Involvement of NLRP3 Inflammasome in the Treatment of Neurodegenerative Diseases. Biomed. Pharmacother. 138, 111428. 10.1016/j.biopha.2021.111428 33667787

[B41] Fink S. L. Cookson B. T. (2006). Caspase-1-dependent Pore Formation during Pyroptosis Leads to Osmotic Lysis of Infected Host Macrophages. Cell. Microbiol. 8, 1812–1825. 10.1111/j.1462-5822.2006.00751.x 16824040

[B42] Flores J. Noël A. Foveau B. Beauchet O. LeBlanc A. C. (2020). Pre-symptomatic Caspase-1 Inhibitor Delays Cognitive Decline in a Mouse Model of Alzheimer Disease and Aging. Nat. Commun. 11, 4571. 10.1038/s41467-020-18405-9 32917871 PMC7486940

[B43] Flores J. Noël A. Foveau B. Lynham J. Lecrux C. LeBlanc A. C. (2018). Caspase-1 Inhibition Alleviates Cognitive Impairment and Neuropathology in an Alzheimer's Disease Mouse Model. Nat. Commun. 9, 3916. 10.1038/s41467-018-06449-x 30254377 PMC6156230

[B44] Frost S. Robinson L. Rowe C. C. Ames D. Masters C. L. Taddei K. (2017). Evaluation of Cholinergic Deficiency in Preclinical Alzheimer's Disease Using Pupillometry. J. Ophthalmol. 2017, 7935406. 10.1155/2017/7935406 28894607 PMC5574262

[B45] Fulp J. He L. Toldo S. Jiang Y. Boice A. Guo C. (2018). Structural Insights of Benzenesulfonamide Analogues as NLRP3 Inflammasome Inhibitors: Design, Synthesis, and Biological Characterization. J. Med. Chem. 61, 5412–5423. 10.1021/acs.jmedchem.8b00733 29877709 PMC6225534

[B46] Gao R. F. Li X. Xiang H. Y. Yang H. Lv C. Y. Sun X. L. (2021a). The Covalent NLRP3-Inflammasome Inhibitor Oridonin Relieves Myocardial Infarction Induced Myocardial Fibrosis and Cardiac Remodeling in Mice. Int. Immunopharmacol. 90, 107133. 10.1016/j.intimp.2020.107133 33168408

[B47] Gao X. Ruan Y. Zhu X. Lin X. Xin Y. Li X. (2021b). Deoxycholic Acid Promotes Pyroptosis in Free Fatty Acid-Induced Steatotic Hepatocytes by Inhibiting PINK1-Mediated Mitophagy. Inflammation. Online. 10.1007/s10753-021-01573-1 34674097

[B48] Gao Y. Li J. Li J. Hu C. Zhang L. Yan J. (2020). Tetrahydroxy Stilbene Glycoside Alleviated Inflammatory Damage by Mitophagy via AMPK Related PINK1/Parkin Signaling Pathway. Biochem. Pharmacol. 177, 113997. 10.1016/j.bcp.2020.113997 32353422

[B49] Ge X. Wang Y. Yu S. Cao X. Chen Y. Cheng Q. (2021). Anti-inflammatory Activity of a Polypeptide Fraction from Achyranthes Bidentate in Amyloid β Oligomers Induced Model of Alzheimer's Disease. Front. Pharmacol. 12, 716177. 10.3389/fphar.2021.716177 34456729 PMC8397449

[B50] Gemma C. Bachstetter A. D. Cole M. J. Fister M. Hudson C. Bickford P. C. (2007). Blockade of Caspase-1 Increases Neurogenesis in the Aged hippocampus. Eur. J. Neurosci. 26, 2795–2803. 10.1111/j.1460-9568.2007.05875.x 18001276

[B51] Gemma C. Fister M. Hudson C. Bickford P. C. (2005). Improvement of Memory for Context by Inhibition of Caspase-1 in Aged Rats. Eur. J. Neurosci. 22, 1751–1756. 10.1111/j.1460-9568.2005.04334.x 16197515

[B52] Giancane G. Minoia F. Davì S. Bracciolini G. Consolaro A. Ravelli A. (2016). IL-1 Inhibition in Systemic Juvenile Idiopathic Arthritis. Front. Pharmacol. 7, 467. 10.3389/fphar.2016.00467 27999545 PMC5138234

[B53] Gu X. Wu H. Xie Y. Xu L. Liu X. Wang W. (2021). Caspase-1/IL-1β Represses Membrane Transport of GluA1 by Inhibiting the Interaction between Stargazin and GluA1 in Alzheimer's Disease. Mol. Med. 27, 8. 10.1186/s10020-021-00273-8 33509083 PMC7842056

[B54] Guan K. Wei C. Zheng Z. Song T. Wu F. Zhang Y. (2015). MAVS Promotes Inflammasome Activation by Targeting ASC for K63-Linked Ubiquitination via the E3 Ligase TRAF3. J. Immunol. 194, 4880–4890. 10.4049/jimmunol.1402851 25847972

[B55] Halle A. Hornung V. Petzold G. C. Stewart C. R. Monks B. G. Reinheckel T. (2008). The NALP3 Inflammasome Is Involved in the Innate Immune Response to Amyloid-Beta. Nat. Immunol. 9, 857–865. 10.1038/ni.1636 18604209 PMC3101478

[B56] Hampel H. Caraci F. Cuello A. C. Caruso G. Nisticò R. Corbo M. (2020). A Path toward Precision Medicine for Neuroinflammatory Mechanisms in Alzheimer's Disease. Front. Immunol. 11, 456. 10.3389/fimmu.2020.00456 32296418 PMC7137904

[B57] Han C. Guo L. Yang Y. Guan Q. Shen H. Sheng Y. (2020a). Mechanism of microRNA-22 in Regulating Neuroinflammation in Alzheimer's Disease. Brain Behav. 10, e01627. 10.1002/brb3.1627 32307887 PMC7303389

[B58] Han C. Yang Y. Guan Q. Zhang X. Shen H. Sheng Y. (2020b). New Mechanism of Nerve Injury in Alzheimer's Disease: β-amyloid-induced Neuronal Pyroptosis. J. Cel. Mol. Med. 24, 8078–8090. 10.1111/jcmm.15439 PMC7348172 32521573

[B59] Han X. Xu T. Fang Q. Zhang H. Yue L. Hu G. (2021). Quercetin Hinders Microglial Activation to Alleviate Neurotoxicity via the Interplay between NLRP3 Inflammasome and Mitophagy. Redox Biol. 44, 102010. 10.1016/j.redox.2021.102010 34082381 PMC8182123

[B60] Hanslik K. L. Ulland T. K. (2020). The Role of Microglia and the Nlrp3 Inflammasome in Alzheimer's Disease. Front. Neurol. 11, 570711. 10.3389/fneur.2020.570711 33071950 PMC7530640

[B61] Hayashi T. Rizzuto R. Hajnoczky G. Su T. P. (2009). MAM: More Than Just a Housekeeper. Trends Cel Biol 19, 81–88. 10.1016/j.tcb.2008.12.002 PMC2750097 19144519

[B62] He H. Jiang H. Chen Y. Ye J. Wang A. Wang C. (2018). Oridonin Is a Covalent NLRP3 Inhibitor with strong Anti-inflammasome Activity. Nat. Commun. 9, 2550. 10.1038/s41467-018-04947-6 29959312 PMC6026158

[B63] He Y. Varadarajan S. Muñoz-Planillo R. Burberry A. Nakamura Y. Núñez G. (2014). 3,4-methylenedioxy-β-nitrostyrene Inhibits NLRP3 Inflammasome Activation by Blocking Assembly of the Inflammasome. J. Biol. Chem. 289, 1142–1150. 10.1074/jbc.M113.515080 24265316 PMC3887181

[B64] Heneka M. T. Kummer M. P. Stutz A. Delekate A. Schwartz S. Vieira-Saecker A. (2013). NLRP3 Is Activated in Alzheimer's Disease and Contributes to Pathology in APP/PS1 Mice. Nature 493, 674–678. 10.1038/nature11729 23254930 PMC3812809

[B65] Hong Y. Liu Y. Yu D. Wang M. Hou Y. (2019). The Neuroprotection of Progesterone against Aβ-Induced NLRP3-Caspase-1 Inflammasome Activation via Enhancing Autophagy in Astrocytes. Int. Immunopharmacol. 74, 105669. 10.1016/j.intimp.2019.05.054 31176046

[B66] Horner S. M. Wilkins C. Badil S. Iskarpatyoti J. Gale M. Jr. (2015). Proteomic Analysis of Mitochondrial-Associated ER Membranes (MAM) during RNA Virus Infection Reveals Dynamic Changes in Protein and Organelle Trafficking. PLoS ONE 10, e0117963. 10.1371/journal.pone.0117963 25734423 PMC4348417

[B67] Horng T. (2014). Calcium Signaling and Mitochondrial Destabilization in the Triggering of the NLRP3 Inflammasome. Trends Immunol. 35, 253–261. 10.1016/j.it.2014.02.007 24646829 PMC4041823

[B68] Huang H. C. Tang D. Lu S. Y. Jiang Z. F. (2015). Endoplasmic Reticulum Stress as a Novel Neuronal Mediator in Alzheimer's Disease. Neurol. Res. 37, 366–374. 10.1179/1743132814Y.0000000448 25310352

[B69] Huang Y. Jiang H. Chen Y. Wang X. Yang Y. Tao J. (2018). Tranilast Directly Targets NLRP3 to Treat Inflammasome-Driven Diseases. EMBO Mol. Med. 10, e8689. 10.15252/emmm.201708689 29531021 PMC5887903

[B70] Ichinohe T. Yamazaki T. Koshiba T. Yanagi Y. (2013). Mitochondrial Protein Mitofusin 2 Is Required for NLRP3 Inflammasome Activation after RNA Virus Infection. Proc. Natl. Acad. Sci. U S A. 110, 17963–17968. 10.1073/pnas.1312571110 24127597 PMC3816452

[B71] Irrera N. Pizzino G. Calò M. Pallio G. Mannino F. Famà F. (2017). Lack of the Nlrp3 Inflammasome Improves Mice Recovery Following Traumatic Brain Injury. Front. Pharmacol. 8, 459. 10.3389/fphar.2017.00459 28769794 PMC5509758

[B72] Ising C. Venegas C. Zhang S. Scheiblich H. Schmidt S. V. Vieira-Saecker A. (2019). NLRP3 Inflammasome Activation Drives Tau Pathology. Nature 575, 669–673. 10.1038/s41586-019-1769-z 31748742 PMC7324015

[B73] Islam B. U. Jabir N. R. Tabrez S. (2019). The Role of Mitochondrial Defects and Oxidative Stress in Alzheimer's Disease. J. Drug Target. 27, 932–942. 10.1080/1061186X.2019.1584808 30775938

[B74] Iyer S. S. He Q. Janczy J. R. Elliott E. I. Zhong Z. Olivier A. K. (2013). Mitochondrial Cardiolipin Is Required for Nlrp3 Inflammasome Activation. Immunity 39, 311–323. 10.1016/j.immuni.2013.08.001 23954133 PMC3779285

[B75] Jiang H. He H. Chen Y. Huang W. Cheng J. Ye J. (2017). Identification of a Selective and Direct NLRP3 Inhibitor to Treat Inflammatory Disorders. J. Exp. Med. 214, 3219–3238. 10.1084/jem.20171419 29021150 PMC5679172

[B76] Juliana C. Fernandes-Alnemri T. Wu J. Datta P. Solorzano L. Yu J. W. (2010). Anti-inflammatory Compounds Parthenolide and Bay 11-7082 Are Direct Inhibitors of the Inflammasome. J. Biol. Chem. 285, 9792–9802. 10.1074/jbc.M109.082305 20093358 PMC2843228

[B77] Jun-An Wang J. A. Tong M. L. Zhao B. Zhu G. Xi D. H. Yang J. P. (2020). Parthenolide Ameliorates Intracerebral Hemorrhage-Induced Brain Injury in Rats. Phytother Res. 34, 153–160. 10.1002/ptr.6510 31497910

[B78] Kajitani N. Iwata M. Miura A. Tsunetomi K. Yamanashi T. Matsuo R. (2020). Prefrontal Cortex Infusion of Beta-Hydroxybutyrate, an Endogenous NLRP3 Inflammasome Inhibitor, Produces Antidepressant-like Effects in a Rodent Model of Depression. Neuropsychopharmacol. Rep. 40, 157–165. 10.1002/npr2.12099 32125791 PMC7722664

[B79] Karkhah A. Saadi M. Pourabdolhossein F. Saleki K. Nouri H. R. (2021). Indomethacin Attenuates Neuroinflammation and Memory Impairment in an STZ-Induced Model of Alzheimer's like Disease. Immunopharmacol Immunotoxicol 43, 758–766. 10.1080/08923973.2021.1981374 34585992

[B80] Kelley N. Jeltema D. Duan Y. He Y. (2019). The NLRP3 Inflammasome: An Overview of Mechanisms of Activation and Regulation. Int. J. Mol. Sci. 20, 3328. 10.3390/ijms20133328 PMC6651423 31284572

[B81] Kim J. Lee H.-j. Park S. K. Park J.-H. Jeong H.-R. Lee S. (2021). Donepezil Regulates LPS and Aβ-Stimulated Neuroinflammation through MAPK/NLRP3 Inflammasome/STAT3 Signaling. Ijms 22, 10637. 10.3390/ijms221910637 34638977 PMC8508964

[B82] Kim N. Do J. Ju I. G. Jeon S. H. Lee J. K. Oh M. S. (2020). Picrorhiza Kurroa Prevents Memory Deficits by Inhibiting NLRP3 Inflammasome Activation and BACE1 Expression in 5xFAD Mice. Neurotherapeutics 17, 189–199. 10.1007/s13311-019-00792-7 31741224 PMC7007473

[B83] Kim S. M. Ha J. S. Han A. R. Cho S. W. Yang S. J. (2019). Effects of α-lipoic Acid on LPS-Induced Neuroinflammation and NLRP3 Inflammasome Activation through the Regulation of BV-2 Microglial Cells Activation. BMB Rep. 52, 613–618. 10.5483/bmbrep.2019.52.10.026 30940325 PMC6827572

[B84] Kinney J. W. Bemiller S. M. Murtishaw A. S. Leisgang A. M. Salazar A. M. Lamb B. T. (2018). Inflammation as a central Mechanism in Alzheimer's Disease. Alzheimers Dement (N Y) 4, 575–590. 10.1016/j.trci.2018.06.014 30406177 PMC6214864

[B85] Kitazawa M. Cheng D. Tsukamoto M. R. Koike M. A. Wes P. D. Vasilevko V. (2011). Blocking IL-1 Signaling Rescues Cognition, Attenuates Tau Pathology, and Restores Neuronal β-catenin Pathway Function in an Alzheimer's Disease Model. J. Immunol. 187, 6539–6549. 10.4049/jimmunol.1100620 22095718 PMC4072218

[B86] Klück V. Jansen T. L. T. A. Janssen M. Comarniceanu A. Efdé M. Tengesdal I. W. (2020). Dapansutrile, an Oral Selective NLRP3 Inflammasome Inhibitor, for Treatment of Gout Flares: an Open-Label, Dose-Adaptive, Proof-Of-Concept, Phase 2a Trial. Lancet Rheumatol. 2, e270–270e280. 10.1016/s2665-9913(20)30065-5 33005902 PMC7523621

[B87] Ko J. W. Shin N. R. Je-Oh L. Jung T. Y. Moon C. Kim T. W. (2020). Silica Dioxide Nanoparticles Aggravate Airway Inflammation in an Asthmatic Mouse Model via NLRP3 Inflammasome Activation. Regul. Toxicol. Pharmacol. 112, 104618. 10.1016/j.yrtph.2020.104618 32087352

[B88] Kumar S. Chang Y. C. Lai K. H. Hwang T. L. (2021). Resveratrol, a Molecule with Anti-inflammatory and Anti-cancer Activities: Natural Product to Chemical Synthesis. Curr. Med. Chem. 28, 3773–3786. 10.2174/0929867327999200918100746 32957870

[B89] Kuwar R. Rolfe A. Di L. Blevins H. Xu Y. Sun X. (2021). A Novel Inhibitor Targeting NLRP3 Inflammasome Reduces Neuropathology and Improves Cognitive Function in Alzheimer's Disease Transgenic Mice. J. Alzheimers Dis. 82, 1769–1783. 10.3233/JAD-210400 34219728 PMC8384725

[B90] Kuwar R. Rolfe A. Di L. Xu H. He L. Jiang Y. (2019). A Novel Small Molecular NLRP3 Inflammasome Inhibitor Alleviates Neuroinflammatory Response Following Traumatic Brain Injury. J. Neuroinflammation 16, 81. 10.1186/s12974-019-1471-y 30975164 PMC6458637

[B91] Lamkanfi M. Dixit V. M. (2014). Mechanisms and Functions of Inflammasomes. Cell 157, 1013–1022. 10.1016/j.cell.2014.04.007 24855941

[B92] Lamkanfi M. Mueller J. L. Vitari A. C. Misaghi S. Fedorova A. Deshayes K. (2009). Glyburide Inhibits the Cryopyrin/Nalp3 Inflammasome. J. Cel Biol. 187, 61–70. 10.1083/jcb.200903124 PMC2762099 19805629

[B93] Lawlor K. E. Vince J. E. (2014). Ambiguities in NLRP3 Inflammasome Regulation: Is There a Role for Mitochondria. Biochim. Biophys. Acta 1840, 1433–1440. 10.1016/j.bbagen.2013.08.014 23994495

[B94] Li D. Yang H. Ma J. Luo S. Chen S. Gu Q. (2018a). MicroRNA-30e regulates neuroinflammation in MPTP model of Parkinson's disease by targeting Nlrp3. Hum. Cel 31, 106–115. 10.1007/s13577-017-0187-5 PMC5852205 29274035

[B95] Li J. Ma C. Long F. Yang D. Liu X. Hu Y. (2019). Parkin Impairs Antiviral Immunity by Suppressing the Mitochondrial Reactive Oxygen Species-Nlrp3 Axis and Antiviral Inflammation. iScience 16, 468–484. 10.1016/j.isci.2019.06.008 31229895 PMC6593176

[B96] Li J. Zhuang L. Luo X. Liang J. Sun E. He Y. (2020a). Protection of MCC950 against Alzheimer's Disease via Inhibiting Neuronal Pyroptosis in SAMP8 Mice. Exp. Brain Res. 238, 2603–2614. 10.1007/s00221-020-05916-6 32892233

[B97] Li K. Wei Q. Liu F. F. Hu F. Xie A. J. Zhu L. Q. (2018b). Synaptic Dysfunction in Alzheimer's Disease: Aβ, Tau, and Epigenetic Alterations. Mol. Neurobiol. 55, 3021–3032. 10.1007/s12035-017-0533-3 28456942

[B98] Li L. Wang X. C. Gong P. T. Zhang N. Zhang X. Li S. (2020b). ROS-mediated NLRP3 Inflammasome Activation Participates in the Response against Neospora Caninum Infection. Parasit Vectors 13, 449. 10.1186/s13071-020-04331-8 32891167 PMC7487665

[B99] Li Q. Chen L. Liu X. Li X. Cao Y. Bai Y. (2018c). Pterostilbene Inhibits Amyloid-β-Induced Neuroinflammation in a Microglia Cell Line by Inactivating the NLRP3/caspase-1 Inflammasome Pathway. J. Cel. Biochem. 119, 7053–7062. 10.1002/jcb.27023 29737568

[B100] Liang H. Sun Y. Gao A. Zhang N. Jia Y. Yang S. (2019). Ac-YVAD-cmk Improves Neurological Function by Inhibiting Caspase-1-Mediated Inflammatory Response in the Intracerebral Hemorrhage of Rats. Int. Immunopharmacol. 75, 105771. 10.1016/j.intimp.2019.105771 31352322

[B101] Lin X. Ye H. Siaw-Debrah F. Pan S. He Z. Ni H. (2018). AC-YVAD-CMK Inhibits Pyroptosis and Improves Functional Outcome after Intracerebral Hemorrhage. Biomed. Res. Int. 2018, 3706047. 10.1155/2018/3706047 30410928 PMC6206581

[B102] Liu P. Huang G. Wei T. Gao J. Huang C. Sun M. (2018). Sirtuin 3-induced Macrophage Autophagy in Regulating NLRP3 Inflammasome Activation. Biochim. Biophys. Acta Mol. Basis Dis. 1864, 764–777. 10.1016/j.bbadis.2017.12.027 29277324

[B103] Liu P. F. Gao T. Li T. Z. Yang Y. T. Xu Y. X. Xu Z. P. (2021). Repeated Propofol Exposure-Induced Neuronal Damage and Cognitive Impairment in Aged Rats by Activation of NF-Κb Pathway and NLRP3 Inflammasome. Neurosci. Lett. 740, 135461. 10.1016/j.neulet.2020.135461 33115643

[B104] Liu X. Hao W. Qin Y. Decker Y. Wang X. Burkart M. (2015). Long-term Treatment with Ginkgo Biloba Extract EGb 761 Improves Symptoms and Pathology in a Transgenic Mouse Model of Alzheimer's Disease. Brain Behav. Immun. 46, 121–131. 10.1016/j.bbi.2015.01.011 25637484

[B105] Liu Y. Dai Y. Li Q. Chen C. Chen H. Song Y. (2020). Beta-amyloid Activates NLRP3 Inflammasome via TLR4 in Mouse Microglia. Neurosci. Lett. 736, 135279. 10.1016/j.neulet.2020.135279 32726591

[B106] Lonnemann N. Hosseini S. Marchetti C. Skouras D. B. Stefanoni D. D'Alessandro A. (2020). The NLRP3 Inflammasome Inhibitor OLT1177 Rescues Cognitive Impairment in a Mouse Model of Alzheimer's Disease. Proc. Natl. Acad. Sci. U S A. 117, 32145–32154. 10.1073/pnas.2009680117 33257576 PMC7749353

[B107] Lučiūnaitė A. McManus R. M. Jankunec M. Rácz I. Dansokho C. Dalgėdienė I. (2020). Soluble Aβ Oligomers and Protofibrils Induce NLRP3 Inflammasome Activation in Microglia. J. Neurochem. 155, 650–661. 10.1111/jnc.14945 31872431

[B108] Luo Z.-L. Sun H.-Y. Wu X.-B. Cheng L. Ren J.-D. (2021). Epigallocatechin-3-gallate Attenuates Acute Pancreatitis Induced Lung Injury by Targeting Mitochondrial Reactive Oxygen Species Triggered NLRP3 Inflammasome Activation. Food Funct. 12, 5658–5667. 10.1039/d1fo01154e 34018522

[B109] Ma X. Li Y. Shen W. Oladejo A. O. Yang J. Jiang W. (2021). LPS Mediates Bovine Endometrial Epithelial Cell Pyroptosis Directly through Both NLRP3 Classical and Non-classical Inflammasome Pathways. Front. Immunol. 12, 676088. 10.3389/fimmu.2021.676088 34122438 PMC8195237

[B110] Mameli E. Martello A. Caporali A. (2021). Autophagy at the Interface of Endothelial Cell Homeostasis and Vascular Disease. FEBS J. (Online) 10.1111/febs.15873 33934518

[B111] Marchetti C. Swartzwelter B. Gamboni F. Neff C. P. Richter K. Azam T. (2018). OLT1177, a β-sulfonyl Nitrile Compound, Safe in Humans, Inhibits the NLRP3 Inflammasome and Reverses the Metabolic Cost of Inflammation. Proc. Natl. Acad. Sci. U S A. 115, E1530–1530E1539. 10.1073/pnas.1716095115 29378952 PMC5816172

[B112] Martinon F. Burns K. Tschopp J. (2002). The Inflammasome: a Molecular Platform Triggering Activation of Inflammatory Caspases and Processing of proIL-Beta. Mol. Cel 10, 417–426. 10.1016/s1097-2765(02)00599-3 12191486

[B113] Martinvalet D. (2018). The Role of the Mitochondria and the Endoplasmic Reticulum Contact Sites in the Development of the Immune Responses. Cell Death Dis 9, 336. 10.1038/s41419-017-0237-7 29491398 PMC5832423

[B114] Minkiewicz J. de Rivero Vaccari J. P. Keane R. W. (2013). Human Astrocytes Express a Novel NLRP2 Inflammasome. Glia 61, 1113–1121. 10.1002/glia.22499 23625868

[B115] Misawa T. Takahama M. Kozaki T. Lee H. Zou J. Saitoh T. (2013). Microtubule-driven Spatial Arrangement of Mitochondria Promotes Activation of the NLRP3 Inflammasome. Nat. Immunol. 14, 454–460. 10.1038/ni.2550 23502856

[B116] Mishra S. R. Mahapatra K. K. Behera B. P. Patra S. Bhol C. S. Panigrahi D. P. (2021). Mitochondrial Dysfunction as a Driver of NLRP3 Inflammasome Activation and its Modulation through Mitophagy for Potential Therapeutics. Int. J. Biochem. Cel Biol. 136, 106013. 10.1016/j.biocel.2021.106013 34022434

[B117] Missiroli S. Patergnani S. Caroccia N. Pedriali G. Perrone M. Previati M. (2018). Mitochondria-associated Membranes (MAMs) and Inflammation. Cel Death Dis 9, 329. 10.1038/s41419-017-0027-2 PMC5832426 29491386

[B118] Murakami T. Ockinger J. Yu J. Byles V. McColl A. Hofer A. M. (2012). Critical Role for Calcium Mobilization in Activation of the NLRP3 Inflammasome. Proc. Natl. Acad. Sci. U S A. 109, 11282–11287. 10.1073/pnas.1117765109 22733741 PMC3396518

[B119] Murphy N. Grehan B. Lynch M. A. (2014). Glial Uptake of Amyloid Beta Induces NLRP3 Inflammasome Formation via Cathepsin-dependent Degradation of NLRP10. Neuromolecular Med. 16, 205–215. 10.1007/s12017-013-8274-6 24197756

[B120] Nakahira K. Haspel J. A. Rathinam V. A. Lee S. J. Dolinay T. Lam H. C. (2011). Autophagy Proteins Regulate Innate Immune Responses by Inhibiting the Release of Mitochondrial DNA Mediated by the NALP3 Inflammasome. Nat. Immunol. 12, 222–230. 10.1038/ni.1980 21151103 PMC3079381

[B121] Niu T. De Rosny C. Chautard S. Rey A. Patoli D. Groslambert M. (2021). NLRP3 Phosphorylation in its LRR Domain Critically Regulates Inflammasome Assembly. Nat. Commun. 12, 5862. 10.1038/s41467-021-26142-w 34615873 PMC8494922

[B122] Osman S. Raza A. Al-Zaidan L. Inchakalody V. P. Merhi M. Prabhu K. S. (2021). Anti-cancer Effects of Tranilast: An Update. Biomed. Pharmacother. 141, 111844. 10.1016/j.biopha.2021.111844 34174504

[B123] Ota M. Matsuo J. Ishida I. Takano H. Yokoi Y. Hori H. (2019). Effects of a Medium-Chain Triglyceride-Based Ketogenic Formula on Cognitive Function in Patients with Mild-To-Moderate Alzheimer's Disease. Neurosci. Lett. 690, 232–236. 10.1016/j.neulet.2018.10.048 30367958

[B124] Ou Y. Sun P. Wu N. Chen H. Wu D. Hu W. (2020). Synthesis and Biological Evaluation of Parthenolide Derivatives with Reduced Toxicity as Potential Inhibitors of the NLRP3 Inflammasome. Bioorg. Med. Chem. Lett. 30, 127399. 10.1016/j.bmcl.2020.127399 32738997

[B125] Panda C. Voelz C. Habib P. Mevissen C. Pufe T. Beyer C. (2021). Aggregated Tau-PHF6 (VQIVYK) Potentiates NLRP3 Inflammasome Expression and Autophagy in Human Microglial Cells. Cells 10, 1652. 10.3390/cells10071652 34209408 PMC8304967

[B126] Peng J. Wang H. Gong Z. Li X. He L. Shen Q. (2020). Idebenone Attenuates Cerebral Inflammatory Injury in Ischemia and Reperfusion via Dampening NLRP3 Inflammasome Activity. Mol. Immunol. 123, 74–87. 10.1016/j.molimm.2020.04.013 32438202

[B127] Peng W. Peng F. Lou Y. Li Y. Zhao N. Shao Q. (2021). Autophagy Alleviates Mitochondrial DAMP-Induced Acute Lung Injury by Inhibiting NLRP3 Inflammasome. Life Sci. 265, 118833. 10.1016/j.lfs.2020.118833 33275990

[B128] Perez-Ruiz F. Chinchilla S. P. Herrero-Beites A. M. (2014). Canakinumab for Gout: a Specific, Patient-Profiled Indication. Expert Rev. Clin. Immunol. 10, 339–347. 10.1586/1744666X.2014.880653 24451032

[B129] Platten M. Wild-Bode C. Wick W. Leitlein J. Dichgans J. Weller M. (2001). N-[3,4-dimethoxycinnamoyl]-anthranilic Acid (Tranilast) Inhibits Transforming Growth Factor-Beta Relesase and Reduces Migration and Invasiveness of Human Malignant Glioma Cells. Int. J. Cancer 93, 53–61. 10.1002/ijc.1289 11391621

[B130] Popugaeva E. Vlasova O. L. Bezprozvanny I. (2015). Restoring Calcium Homeostasis to Treat Alzheimer's Disease: a Future Perspective. Neurodegener Dis. Manag. 5, 395–398. 10.2217/nmt.15.36 26477700 PMC5558533

[B131] Qi Y. Klyubin I. Cuello A. C. Rowan M. J. (2018). NLRP3-dependent Synaptic Plasticity Deficit in an Alzheimer's Disease Amyloidosis Model *In Vivo* . Neurobiol. Dis. 114, 24–30. 10.1016/j.nbd.2018.02.016 29477641

[B132] Qi Y. Shang L. Liao Z. Su H. Jing H. Wu B. (2019). Intracerebroventricular Injection of Resveratrol Ameliorated Aβ-Induced Learning and Cognitive Decline in Mice. Metab. Brain Dis. 34, 257–266. 10.1007/s11011-018-0348-6 30460524

[B133] Qin Y. Qiu J. Wang P. Liu J. Zhao Y. Jiang F. (2021). Impaired Autophagy in Microglia Aggravates Dopaminergic Neurodegeneration by Regulating NLRP3 Inflammasome Activation in Experimental Models of Parkinson's Disease. Brain Behav. Immun. 91, 324–338. 10.1016/j.bbi.2020.10.010 33039664

[B134] Qiu W. Q. Pan R. Tang Y. Zhou X. G. Wu J. M. Yu L. (2020). Lychee Seed Polyphenol Inhibits Aβ-Induced Activation of NLRP3 Inflammasome via the LRP1/AMPK Mediated Autophagy Induction. Biomed. Pharmacother. 130, 110575. 10.1016/j.biopha.2020.110575 32768883

[B135] Ravizza T. Noé F. Zardoni D. Vaghi V. Sifringer M. Vezzani A. (2008). Interleukin Converting Enzyme Inhibition Impairs Kindling Epileptogenesis in Rats by Blocking Astrocytic IL-1beta Production. Neurobiol. Dis. 31, 327–333. 10.1016/j.nbd.2008.05.007 18632279

[B136] Reddy P. H. Oliver D. M. (2019). Amyloid Beta and Phosphorylated Tau-Induced Defective Autophagy and Mitophagy in Alzheimer's Disease. Cells 8, 488. 10.3390/cells8050488 PMC6562604 31121890

[B137] Rimessi A. Bezzerri V. Patergnani S. Marchi S. Cabrini G. Pinton P. (2015). Mitochondrial Ca2+-dependent NLRP3 Activation Exacerbates the Pseudomonas Aeruginosa-Driven Inflammatory Response in Cystic Fibrosis. Nat. Commun. 6, 6201. 10.1038/ncomms7201 25648527

[B138] Ruan Y. Qiu X. Lv Y. D. Dong D. Wu X. J. Zhu J. (2019). Kainic Acid Induces Production and Aggregation of Amyloid β-protein and Memory Deficits by Activating Inflammasomes in NLRP3- and NF-Κb-Stimulated Pathways. Aging (Albany NY) 11, 3795–3810. 10.18632/aging.102017 31182681 PMC6594814

[B139] Saitoh T. Fujita N. Jang M. H. Uematsu S. Yang B. G. Satoh T. (2008). Loss of the Autophagy Protein Atg16L1 Enhances Endotoxin-Induced IL-1beta Production. Nature 456, 264–268. 10.1038/nature07383 18849965

[B140] Sanabria-Castro A. Alvarado-Echeverría I. Monge-Bonilla C. (2017). Molecular Pathogenesis of Alzheimer's Disease: An Update. Ann. Neurosci. 24, 46–54. 10.1159/000464422 28588356 PMC5448443

[B141] Saresella M. La Rosa F. Piancone F. Zoppis M. Marventano I. Calabrese E. (2016). The NLRP3 and NLRP1 Inflammasomes Are Activated in Alzheimer's Disease. Mol. Neurodegener 11, 23. 10.1186/s13024-016-0088-1 26939933 PMC4778358

[B142] Sarkar S. Malovic E. Harishchandra D. S. Ghaisas S. Panicker N. Charli A. (2017). Mitochondrial Impairment in Microglia Amplifies NLRP3 Inflammasome Proinflammatory Signaling in Cell Culture and Animal Models of Parkinson's Disease. NPJ Parkinsons Dis. 3, 30. 10.1038/s41531-017-0032-2 29057315 PMC5645400

[B143] Schroder K. Tschopp J. (2010). The Inflammasomes. Cell 140, 821–832. 10.1016/j.cell.2010.01.040 20303873

[B190] Shi J. Zhao Y. Wang K. Shi X. Wang Y. Huang H. (2015). Cleavage of GSDMD by Inflammatory Caspases Determines Pyroptotic Cell Death. Nature 526, 660–665. 10.1038/nature15514 26375003

[B144] Shimada K. Crother T. R. Karlin J. Dagvadorj J. Chiba N. Chen S. (2012). Oxidized Mitochondrial DNA Activates the NLRP3 Inflammasome during Apoptosis. Immunity 36, 401–414. 10.1016/j.immuni.2012.01.009 22342844 PMC3312986

[B145] Shippy D. C. Wilhelm C. Viharkumar P. A. Raife T. J. Ulland T. K. (2020). β-Hydroxybutyrate Inhibits Inflammasome Activation to Attenuate Alzheimer's Disease Pathology. J. Neuroinflammation 17, 280. 10.1186/s12974-020-01948-5 32958021 PMC7507727

[B146] Song H. Liu B. Huai W. Yu Z. Wang W. Zhao J. (2016). The E3 Ubiquitin Ligase TRIM31 Attenuates NLRP3 Inflammasome Activation by Promoting Proteasomal Degradation of NLRP3. Nat. Commun. 7, 13727. 10.1038/ncomms13727 27929086 PMC5155141

[B147] Sota J. Rigante D. Cimaz R. Cattalini M. Frassi M. Manna R. (2021). Drug Survival of Anakinra and Canakinumab in Monogenic Autoinflammatory Diseases: Observational Study from the International AIDA Registry. Rheumatology (Oxford) 60, 5705–5712. 10.1093/rheumatology/keab419 33961014

[B148] Stancu I. C. Cremers N. Vanrusselt H. Couturier J. Vanoosthuyse A. Kessels S. (2019). Aggregated Tau Activates NLRP3-ASC Inflammasome Exacerbating Exogenously Seeded and Non-exogenously Seeded Tau Pathology *In Vivo* . Acta Neuropathol. 137, 599–617. 10.1007/s00401-018-01957-y 30721409 PMC6426830

[B149] Subramanian N. Natarajan K. Clatworthy M. R. Wang Z. Germain R. N. (2013). The Adaptor MAVS Promotes NLRP3 Mitochondrial Localization and Inflammasome Activation. Cell 153, 348–361. 10.1016/j.cell.2013.02.054 23582325 PMC3632354

[B151] Swanson K. V. Deng M. Ting J. P. (2019). The NLRP3 Inflammasome: Molecular Activation and Regulation to Therapeutics. Nat. Rev. Immunol. 19, 477–489. 10.1038/s41577-019-0165-0 31036962 PMC7807242

[B152] Szabadkai G. Bianchi K. Várnai P. De Stefani D. Wieckowski M. R. Cavagna D. (2006). Chaperone-mediated Coupling of Endoplasmic Reticulum and Mitochondrial Ca2+ Channels. J. Cel Biol. 175, 901–911. 10.1083/jcb.200608073 PMC2064700 17178908

[B153] Szabo L. Eckert A. Grimm A. (2020). Insights into Disease-Associated Tau Impact on Mitochondria. Int. J. Mol. Sci. 21, 6344. 10.3390/ijms21176344 PMC7503371 32882957

[B154] Tang T. Li P. Zhou X. Wang R. Fan X. Yang M. (2021). The E3 Ubiquitin Ligase TRIM65 Negatively Regulates Inflammasome Activation through Promoting Ubiquitination of NLRP3. Front. Immunol. 12, 741839. 10.3389/fimmu.2021.741839 34512673 PMC8427430

[B155] Tapia-Abellán A. Angosto-Bazarra D. Martínez-Banaclocha H. de Torre-Minguela C. Cerón-Carrasco J. P. Pérez-Sánchez H. (2019). MCC950 Closes the Active Conformation of NLRP3 to an Inactive State. Nat. Chem. Biol. 15, 560–564. 10.1038/s41589-019-0278-6 31086329 PMC7116292

[B156] Terrill-Usery S. E. Mohan M. J. Nichols M. R. (2014). Amyloid-β(1-42) Protofibrils Stimulate a Quantum of Secreted IL-1β Despite Significant Intracellular IL-1β Accumulation in Microglia. Biochim. Biophys. Acta 1842, 2276–2285. 10.1016/j.bbadis.2014.08.001 25125050 PMC4188733

[B157] Thapak P. Bishnoi M. Sharma S. S. (2021). Tranilast, a Transient Receptor Potential Vanilloid 2 Channel (TRPV2) Inhibitor Attenuates Amyloid β-Induced Cognitive Impairment: Possible Mechanisms. Neuromol Med. (Online). 10.1007/s12017-021-08675-x 34231190

[B158] Tufekci K. U. Ercan I. Isci K. B. Olcum M. Tastan B. Gonul C. P. (2021). Sulforaphane Inhibits NLRP3 Inflammasome Activation in Microglia through Nrf2-Mediated miRNA Alteration. Immunol. Lett. 233, 20–30. 10.1016/j.imlet.2021.03.004 33711331

[B159] Van Zeller M. Dias D. Sebastião A. M. Valente C. A. (2021). NLRP3 Inflammasome: A Starring Role in Amyloid-β- and Tau-Driven Pathological Events in Alzheimer's Disease. J. Alzheimers Dis. 83, 939–961. 10.3233/JAD-210268 34366341 PMC8543248

[B160] von Herrmann K. M. Salas L. A. Martinez E. M. Young A. L. Howard J. M. Feldman M. S. (2018). NLRP3 Expression in Mesencephalic Neurons and Characterization of a Rare NLRP3 Polymorphism Associated with Decreased Risk of Parkinson's Disease. NPJ Parkinsons Dis. 4, 24. 10.1038/s41531-018-0061-5 30131971 PMC6093937

[B161] Wan S. Y. Li G. S. Tu C. Chen W. L. Wang X. W. Wang Y. N. (2021). MicroNAR-194-5p Hinders the Activation of NLRP3 Inflammasomes and Alleviates Neuroinflammation during Intracerebral Hemorrhage by Blocking the Interaction between TRAF6 and NLRP3. Brain Res. 1752, 147228. 10.1016/j.brainres.2020.147228 33385377

[B150] Wang S. Yang H. Yu L. Jin J. Qian L. Zhao H. (2014). Oridonin Attenuates Aβ1-42-Induced Neuroinflammation and Inhibits NF-Κb Pathway. PLoS ONE 9, e104745. 10.1371/journal.pone.0104745 25121593 PMC4133239

[B162] Wang C. Y. Xu Y. Wang X. Guo C. Wang T. Wang Z. Y. (2019). Dl-3-n-Butylphthalide Inhibits NLRP3 Inflammasome and Mitigates Alzheimer's-like Pathology via Nrf2-TXNIP-TrX Axis. Antioxid. Redox Signal. 30, 1411–1431. 10.1089/ars.2017.7440 29634349

[B163] Wang H. M. Zhang T. Huang J. K. Xiang J. Y. Chen J. J. Fu J. L. (2017). Edaravone Attenuates the Proinflammatory Response in Amyloid-β-Treated Microglia by Inhibiting NLRP3 Inflammasome-Mediated IL-1β Secretion. Cel. Physiol. Biochem. 43, 1113–1125. 10.1159/000481753 28977782

[B164] Wang J. Z. Wang Z. H. Tian Q. (2014). Tau Hyperphosphorylation Induces Apoptotic Escape and Triggers Neurodegeneration in Alzheimer's Disease. Neurosci. Bull. 30, 359–366. 10.1007/s12264-013-1415-y 24627329 PMC5562660

[B165] Wang S. Yu L. Yang H. Li C. Hui Z. Xu Y. (2016). Oridonin Attenuates Synaptic Loss and Cognitive Deficits in an Aβ1-42-Induced Mouse Model of Alzheimer's Disease. PLoS ONE 11, e0151397. 10.1371/journal.pone.0151397 26974541 PMC4790895

[B166] Wang W. Y. Hsieh P. W. Wu Y. C. Wu C. C. (2007). Synthesis and Pharmacological Evaluation of Novel Beta-Nitrostyrene Derivatives as Tyrosine Kinase Inhibitors with Potent Antiplatelet Activity. Biochem. Pharmacol. 74, 601–611. 10.1016/j.bcp.2007.06.001 17601492

[B167] Wang X. Chi J. Huang D. Ding L. Zhao X. Jiang L. (2020). α-Synuclein Promotes Progression of Parkinson's Disease by Upregulating Autophagy Signaling Pathway to Activate NLRP3 Inflammasome. Exp. Ther. Med. 19, 931–938. 10.3892/etm.2019.8297 32010254 PMC6966172

[B168] Wang X. Sun K. Zhou Y. Wang H. Zhou Y. Liu S. (2021). NLRP3 Inflammasome Inhibitor CY-09 Reduces Hepatic Steatosis in Experimental NAFLD Mice. Biochem. Biophys. Res. Commun. 534, 734–739. 10.1016/j.bbrc.2020.11.009 33213837

[B169] Wen M. Ding L. Zhang L. Zhang T. Teruyoshi Y. Wang Y. (2019). Eicosapentaenoic Acid-Enriched Phosphatidylcholine Mitigated Aβ1-42-Induced Neurotoxicity via Autophagy-Inflammasome Pathway. J. Agric. Food Chem. 67, 13767–13774. 10.1021/acs.jafc.9b05947 31722531

[B170] Xu F. Qi H. Li J. Sun L. Gong J. Chen Y. (2020). Mycobacterium tuberculosis Infection Upregulates MFN2 Expression to Promote NLRP3 Inflammasome Formation. J. Biol. Chem. 295, 17684–17697. 10.1074/jbc.RA120.014077 33454007 PMC7762945

[B171] Xu L. Wang Q. Jiang W. Yu S. Zhang S. (2019). MiR-34c Ameliorates Neuropathic Pain by Targeting NLRP3 in a Mouse Model of Chronic Constriction Injury. Neuroscience 399, 125–134. 10.1016/j.neuroscience.2018.12.030 30593918

[B172] Yamanashi T. Iwata M. Kamiya N. Tsunetomi K. Kajitani N. Wada N. (2017). Beta-hydroxybutyrate, an Endogenic NLRP3 Inflammasome Inhibitor, Attenuates Stress-Induced Behavioral and Inflammatory Responses. Sci. Rep. 7, 7677. 10.1038/s41598-017-08055-1 28794421 PMC5550422

[B173] Yamanashi T. Iwata M. Shibushita M. Tsunetomi K. Nagata M. Kajitani N. (2020). Beta-hydroxybutyrate, an Endogenous NLRP3 Inflammasome Inhibitor, Attenuates Anxiety-Related Behavior in a Rodent post-traumatic Stress Disorder Model. Sci. Rep. 10, 21629. 10.1038/s41598-020-78410-2 33303808 PMC7728809

[B174] Yan C. Yan H. Mao J. Liu Y. Xu L. Zhao H. (2020a). Neuroprotective Effect of Oridonin on Traumatic Brain Injury via Inhibiting NLRP3 Inflammasome in Experimental Mice. Front. Neurosci. 14, 557170. 10.3389/fnins.2020.557170 33281541 PMC7691250

[B175] Yan S. Xuan Z. Yang M. Wang C. Tao T. Wang Q. (2020b). CSB6B Prevents β-amyloid-associated Neuroinflammation and Cognitive Impairments via Inhibiting NF-Κb and NLRP3 in Microglia Cells. Int. Immunopharmacol. 81, 106263. 10.1016/j.intimp.2020.106263 32028243

[B176] Yang Y. Wang H. Kouadir M. Song H. Shi F. (2019). Recent Advances in the Mechanisms of NLRP3 Inflammasome Activation and its Inhibitors. Cel Death Dis 10, 128. 10.1038/s41419-019-1413-8 PMC6372664 30755589

[B177] Yin J. Zhao F. Chojnacki J. E. Fulp J. Klein W. L. Zhang S. (2018). NLRP3 Inflammasome Inhibitor Ameliorates Amyloid Pathology in a Mouse Model of Alzheimer's Disease. Mol. Neurobiol. 55, 1977–1987. 10.1007/s12035-017-0467-9 28255908 PMC5585057

[B178] Youdim K. A. Dobbie M. S. Kuhnle G. Proteggente A. R. Abbott N. J. Rice-Evans C. (2003). Interaction between Flavonoids and the Blood-Brain Barrier: *In Vitro* Studies. J. Neurochem. 85, 180–192. 10.1046/j.1471-4159.2003.01652.x 12641740

[B179] Youm Y. H. Nguyen K. Y. Grant R. W. Goldberg E. L. Bodogai M. Kim D. (2015). The Ketone Metabolite β-hydroxybutyrate Blocks NLRP3 Inflammasome-Mediated Inflammatory Disease. Nat. Med. 21, 263–269. 10.1038/nm.3804 25686106 PMC4352123

[B180] Yu W. Jin H. Huang Y. (2021). Mitochondria-associated Membranes (MAMs): a Potential Therapeutic Target for Treating Alzheimer's Disease. Clin. Sci. 135, 109–126. 10.1042/CS20200844 PMC7796309 33404051

[B181] Zhang M. Wang L. Huang S. He X. (2020). MicroRNA-223 Targets NLRP3 to Relieve Inflammation and Alleviate Spinal Cord Injury. Life Sci. 254, 117796. 10.1016/j.lfs.2020.117796 32417375

[B182] Zhang Y. Lin Z. Chen D. He Y. (2021a). CY-09 Attenuates the Progression of Osteoarthritis via Inhibiting NLRP3 Inflammasome-Mediated Pyroptosis. Biochem. Biophys. Res. Commun. 553, 119–125. 10.1016/j.bbrc.2021.03.055 33765556

[B183] Zhang Y. Zhao Y. Zhang J. Gao Y. Li S. Chang C. (2021b). Ginkgolide B Inhibits NLRP3 Inflammasome Activation and Promotes Microglial M2 Polarization in Aβ1-42-Induced Microglia Cells. Neurosci. Lett. 764, 136206. 10.1016/j.neulet.2021.136206 34478813

[B184] Zhang Y. Zhao Y. Zhang J. Yang G. (2020). Mechanisms of NLRP3 Inflammasome Activation: Its Role in the Treatment of Alzheimer's Disease. Neurochem. Res. 45, 2560–2572. 10.1007/s11064-020-03121-z 32929691

[B185] Zhao Y. Tan S. W. Huang Z. Z. Shan F. B. Li P. Ning Y. L. (2021). NLRP3 Inflammasome-dependent Increases in High Mobility Group Box 1 Involved in the Cognitive Dysfunction Caused by Tau-Overexpression. Front. Aging Neurosci. 13, 721474. 10.3389/fnagi.2021.721474 34539383 PMC8446370

[B186] Zhong Z. Liang S. Sanchez-Lopez E. He F. Shalapour S. Lin X. J. (2018). New Mitochondrial DNA Synthesis Enables NLRP3 Inflammasome Activation. Nature 560, 198–203. 10.1038/s41586-018-0372-z 30046112 PMC6329306

[B187] Zhou R. Yazdi A. S. Menu P. Tschopp J. (2011). A Role for Mitochondria in NLRP3 Inflammasome Activation. Nature 469, 221–225. 10.1038/nature09663 21124315

[B188] Zhou Y. Lu M. Du R. H. Qiao C. Jiang C. Y. Zhang K. Z. (2016). MicroRNA-7 Targets Nod-like Receptor Protein 3 Inflammasome to Modulate Neuroinflammation in the Pathogenesis of Parkinson's Disease. Mol. Neurodegener 11, 28. 10.1186/s13024-016-0094-3 27084336 PMC4833896

[B189] Zhu T. B. Zhang Z. Luo P. Wang S. S. Peng Y. Chu S. F. (2019). Lipid Metabolism in Alzheimer's Disease. Brain Res. Bull. 144, 68–74. 10.1016/j.brainresbull.2018.11.012 30472149

